# Photoactivable Ruthenium-Based Coordination Polymer Nanoparticles for Light-Induced Chemotherapy

**DOI:** 10.3390/nano11113089

**Published:** 2021-11-16

**Authors:** Junda Zhang, Vadde Ramu, Xue-Quan Zhou, Carolina Frias, Daniel Ruiz-Molina, Sylvestre Bonnet, Claudio Roscini, Fernando Novio

**Affiliations:** 1 Catalan Institute of Nanoscience and Nanotechnology (ICN2), CSIC and BIST, Campus UAB, Bellaterra, 08193 Barcelona, Spain; junda.zhang@icn2.cat (J.Z.); caarolina.frias@gmail.com (C.F.); dani.ruiz@icn2.cat (D.R.-M.); 2 Leiden Institute of Chemistry, Universiteit Leiden, Einsteinweg 55, 2333 CC Leiden, The Netherlands; ramuvadde1@gmail.com (V.R.); x.zhou@lic.leidenuniv.nl (X.-Q.Z.)

**Keywords:** coordination polymer nanoparticles, nanoparticles, photoactivated chemotherapy, prodrug, drug delivery, ruthenium-based drug

## Abstract

Green light photoactive Ru-based coordination polymer nanoparticles (CPNs), with chemical formula [[Ru(biqbpy)]_1.5_(bis)](PF_6_)_3_ (biqbpy = 6,6′-bis[N-(isoquinolyl)-1-amino]-2,2′-bipyridine; bis = bis(imidazol-1-yl)-hexane), were obtained through polymerization of the trans-[Ru(biqbpy)(dmso)Cl]Cl complex (Complex **1**) and bis bridging ligands. The as-synthesized CPNs (50 ± 12 nm diameter) showed high colloidal and chemical stability in physiological solutions. The axial bis(imidazole) ligands coordinated to the ruthenium center were photosubstituted by water upon light irradiation in aqueous medium to generate the aqueous substituted and active ruthenium complexes. The UV-Vis spectral variations observed for the suspension upon irradiation corroborated the photoactivation of the CPNs, while High Performance Liquid Chromatography (HPLC) of irradiated particles in physiological media allowed for the first time precisely quantifying the amount of photoreleased complex from the polymeric material. In vitro studies with A431 and A549 cancer cell lines revealed an 11-fold increased uptake for the nanoparticles compared to the monomeric complex [Ru(biqbpy)(N-methylimidazole)_2_](PF_6_)_2_ (Complex **2**). After irradiation (520 nm, 39.3 J/cm^2^), the CPNs yielded up to a two-fold increase in cytotoxicity compared to the same CPNs kept in the dark, indicating a selective effect by light irradiation. Meanwhile, the absence of ^1^O_2_ production from both nanostructured and monomeric prodrugs concluded that light-induced cell death is not caused by a photodynamic effect but rather by photoactivated chemotherapy.

## 1. Introduction

Ruthenium-based drugs have raised interest over the last years as an alternative to Pt drugs for oncotherapy, with an increasing number of them entering clinical trials, such as NAMI-A, KP 1019, KP 1339, or TLD–1433 [1,2,3,4,5,6,7]. Especially relevant has been the development of ruthenium prodrug molecular complexes bearing photolabile ligands for photoactivated chemotherapy (PACT) applications [8,9,10,11,12]. Interestingly, these complexes generally exhibit low toxicity in the dark but become toxic once activated by visible light irradiation. The mechanism of prodrug activation is related to the specific ligand photosubstitution by water molecules, to afford activated aqua photoproducts able to induce a therapeutic action [10,13,14,15,16,17]. Moreover, PACT is an oxygen-independent activation mechanism that works even under hypoxic conditions. This feature makes it potentially more versatile than type II photodynamic therapy (PDT), which requires the presence of a significant amount of dioxygen to generate enough reactive singlet oxygen species to induce cytotoxicity [10,11,14,18,19,20,21,22,23,24].

However, before reaching clinical use, photoactivated Ru complexes must face key challenges such as water solubility, preferential accumulation in tumors [25], precise controlled release of the drug [26,27,28], increase in the biocompatibility while minimizing residual toxicity in the dark [29,30,31], and improvement of their fast clearance from the bloodstream [32]. To overcome most of these limitations, photolabile complexes [33,34], and specifically Ru-based complexes [35], can be incorporated in nanoparticles (NPs) for their application in photoinduced therapies. For instance, Wu et al. have reported the covalent link of Ru to block copolymers [10,36] that stabilize photoactivatable ruthenium complexes under physiological conditions [9]. This strategy includes ruthenium-containing block copolymer units that self-assembled into nanoparticles in aqueous solution with excellent uptake in vitro and in vivo results. The inhibition of cancer cells was related to the generation of singlet oxygen (^1^O_2_) upon irradiation with red light [37,38]. Other approaches involve the conjugation of photocleavable Ru complexes to the surface of upconverting NPs [39,40,41,42]. Even so, the encapsulation of photoactive Ru-based complexes is in its fledgling stage, so there is a growing interest to develop novel NPs that allow a proper fine-tune structure/function correlations and adapt it for their use in photoactivated chemotherapy [43].

**Figure 1:**
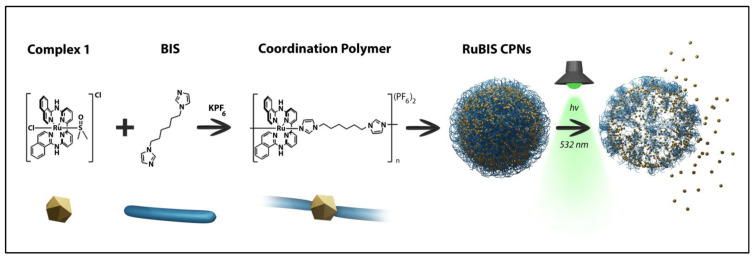
Scheme of synthesis and photoactivation process of Ru-based coordination polymer nanoparticles (**RuBIS** CPNs).

Herein, we hypothesize that coordination polymer nanoparticles (CPNs) bearing Ru-active complexes as constitutive building blocks and a photocleavable bridging ligand may represent a step forward for PACT applications (the schematic representation of the proposed system is shown in Figure 1). In addition to achieve smart NPs with high payloads, CPNs have already been successfully demonstrated to be highly performing as biocompatible contrast agents and antitumoral application, including Pt (IV)-based CPNs [44,45]. These nanoformulations offer good colloidal stability, scalability, cellular internalization, and even more noticeably high payloads, as the prodrug constitutes the backbone of the nanoparticles polymer itself [46]. All these advantages turn out to be really helpful to reduce the dose, the irradiation intensity required to activate the anticancer drug diffusion, and therefore any side effect. Though, as far as we know, the number of ruthenium-based coordination polymers with antitumor applications is rather limited, none of them being photoactivable as far as we know.

We have achieved this challenge with the synthesis of CPNs containing [Ru(biqbpy)(dmso)Cl]Cl monomer (complex **1**, where biqbpy = stands for 6,6′-*bis*[N-(isoquinolyl)-1-amino]-2,2′-bipyridine), which is known to form cytotoxic aqueous active species [Ru(biqbpy)(H_2_O)_2_]^2+^ upon blue or green light activation [14]. The polymerization process was performed using the photocleavable *bis*(imidazol-1-yl)-hexane (**BIS**) ligand and following a methodology previously described for the synthesis of non-photoactive CPNs of relevance in biological applications [47,48,49,50].

## 2. Materials and Methods

### 2.1. Reagents and Instrumentation

Solvents were purchased from Sigma–Aldrich (Merck KGaA, Darmstadt, Alemania) and used as received, and complex **1** was synthesized and characterized according to previously reported methodology [14]. Fourier transform infrared (FTIR) spectra were carried out with a Tensor 27/PMA50FTIR Spectrometer (Bruker Optics GmbH, Ettlingen, Germany) in a range of 4000–400 cm^−1^. Determination of the particle-size distributions and the zeta potential values were measured by dynamic light scattering (DLS) using a ZetaSizer nano ZS (ZEN3600, Malvern Instruments, Ltd., Malvern, UK). Scanning Electron Microscopy (SEM) images were obtained on a scanning electron microscope (FEI Quanta 650 FEG, Thermo Fisher Scientific, Eindhoven, The Netherlands). The samples were casted on aluminum holders following by evaporation, and later, a thin platinum layer was sprayed to increase the conductivity of samples. Ultraviolet–visible spectroscopy (UV-vis) study was carried out in the Agilent Cary 60 spectrophotometer (Agilent Technologies, Santa Clara, CA, USA) in the dark using a 1 cm quartz cuvette with a stirring bar, containing 2 mL of **RuBIS** CPNs (20 μg/mL). Time-dependent UV-Vis spectra during irradiation were recorded at regular time intervals (specified in the spectra) after irradiation of the stirred sample with a continuous beam of a green (λ = 532 nm, 30 mW, 0.42 mW/cm^2^) or blue laser (450 nm, 100 mW, 0.35 mW/cm^2^) set in front of the cuvette.

### 2.2. Synthesis and Characterization of the Photoactive Materials

#### 2.2.1. Synthesis of Complex **2**

**Figure 2:**
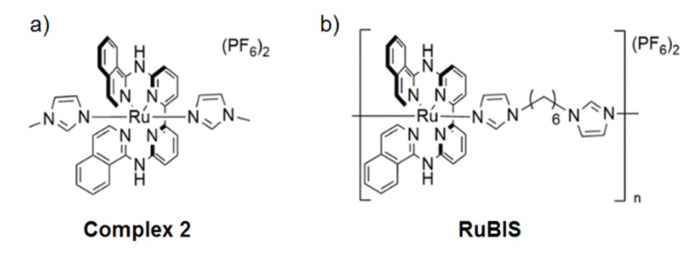
Chemical representation of complex **2** (**a**) and **RuBIS** CPNs (**b**).

[Ru(biqbpy)(dmso)Cl]Cl (50.0 mg, 0.072 mmol) was dissolved in EtOH and H_2_O (5 mL, *v/v* = 1/1). Then, 1-methylimidazole (60.0 mg, 0.73 mmol) was injected in the mixture and refluxed for 12 h under N_2_ protection. The reaction mixture was cooled to room temperature. Then, ice-cold water (5 mL) and KPF_6_ (20 mg) were added to the reaction mixture; thus, dark brown precipitate was formed and filtered. After column chromatography (SiO_2_, Ethyl acetate/MeOH = 10:1), 2 (50 mg, 80%) [Ru(biqbpy)(N-methylimidazole)_2_] (PF_6_)_2_ complex (complex **2**, Figure 2a) was obtained as a dark brown solid. For NMR and mass spectrometry characterization, see Figures S1–S4.

^1^H NMR (360 MHz, (CD_3_)_2_SO) δ 10.73 (s, 2H), 8.93 (d, J = 8.1 Hz, 2H), 8.46 (d, J = 7.7 Hz, 2H), 8.21 (d, J = 6.7 Hz, 2H), 8.12 (t, J = 8.0 Hz, 2H), 7.93 (ddd, J = 23.1, 18.0, 7.3 Hz, 8H), 7.41 (d, J = 6.6 Hz, 2H), 7.32 (s, 2H), 6.86 (s, 2H), 6.08 (s, 2H), 3.35 (s, 6H). ^13^C NMR (91 MHz, (CD_3_)_2_SO) δ 156.28, 151.20, 150.01, 145.50, 140.25, 135.81, 135.58, 131.80, 129.28, 128.82, 127.86, 123.77, 122.69, 120.21, 118.81, 116.06, 115.79, 34.22. MS-ESI (*m*/*z*): [M]+ Calcd. for C_36_H_32_N_10_Ru^+^ 705.8, found 705.2. Elem. Anal. Calcd. for C_36_H_32_F_12_N_10_P_2_Ru·3H_2_O: C, 40.55; H, 3.60; N, 13.51 Found: C, 40.72; H, 3.62; N, 12.95.

#### 2.2.2. Synthesis of Coordination Polymer Nanoparticles RuBIS 

Ru complex ([Ru(biqbpy)(dmso)Cl]Cl) (10.9 mg, 15.8 μmol) was firstly dissolved in a 2-necked round-bottom flask (10 mL) in 1 mL of Milli-Q^®^ water under reflux and N_2_ atmosphere for 10 min. **BIS** (3.4 mg, 15.8 μmol) was dissolved in 1 mL of Milli-Q^®^ water and injected to the reaction slowly, and the color of the reaction changed from orange to dark brown. The reaction was stirred at 80 °C at 600 rpm for 1 h in dark conditions. After 1 h, 0.5 mL of saturated KPF_6_ water solution was added to the mixture, causing the precipitation of a solid. The solid was purified through three times centrifugation (10 min, 4300 rpm) and washed with Milli-Q^®^ water. Finally, the as-obtained solid was freeze-dried and stored as a powder (13.0 mg, yield = 92.8 wt %). Chemical analysis, detailed in the results and discussion section, enabled us to propose the chemical formula [[Ru(biqbpy)]_1.5_(bis)](PF_6_)_3_ (Figure 2b).

### 2.3. HPLC Methodology for RuBIS CPNs Releasing Quantification

Time-dependent HPLC evolution under irradiation of a **RuBIS** colloidal suspension (200 µg/mL) was performed with stirring, and aliquots at different irradiation times were taken, filtered, and analyzed by HPLC. A calibration curve was performed using different concentrations (0.1, 0.5, 1, 5, 10, and 20 μg/mL) of a stock solution in PBS buffer of the [Ru(biqbpy)(H_2_O)_2_]^2+^ activated complex. The stock solution was obtained from the irradiation with green light (100 mW, 1.1 mW/cm^2^) of complex **1** (1 mg/mL) dissolved in PBS solution for 20 h to make sure that Ru complex was fully converted to the active form through the photocleavage and photosubstitution process. The measurements were carried out using an HPLC Waters 2695 separation module (Waters Corp., Milford, MA, USA) coupled to a Waters 2487 UV-Vis detector (Waters Corp., Milford, MA, USA) and using a Restek^®^ C-18 (250 mm × 4.6 mm) column (Restek Corp., Bellefonte, PA, USA). Eluent A was a 0.1% (*v*/*v*) H_3_PO_4_ aqueous solution, and eluent B was acetonitrile absolute (HPLC grade). Before the analysis, the column was pre-equilibrated using the starting conditions (99% A (*v*/*v*)) for 10 min, followed by a gradual decrease in A from 100% to 40% (*v*/*v*) in the first 20 min and lasting 5 min. Then, the mobile phase reduced to 20% A (*v*/*v*) in 1 min and lasted 4 min. At the end, mobile phase was increased to 100% A (*v*/*v*) in 1 min to elute residues, and this ratio was kept for additional 5 min. For the next injection, the mobile phase was reset to the initial conditions (A:B) 100:0 (*v*/*v*) and kept for 10 min to equilibrate. The flow rate was set at 1.0 mL/min at temperature. This method was used for both the calibration curve and quantification of an active complex release from **RuBIS** CPNs.

### 2.4. Quantitative ^1^H NMR and ^19^F NMR for Component Analysis of RuBIS CPNs

The slow rotational correlation time in NMR of these nanoparticles in a colloidal solution makes it difficult to obtain a quantitative NMR spectrum. To accomplish this, the **RuBIS** CPNs were dissolved in deuterated dimethyl sulfoxide solvent ((CD_3_)_2_SO) containing a minimum quantity of deuterium chloride (DCl) to decompose the nanoparticles into the molecular entities and thus obtain sharp signals that allow quantifying the ligand-to-ligand ratio. To make sure that different spectra are comparable, the same ratio DCl/(CD_3_)_2_SO was used (50 µL DCl/mL (CD_3_)_2_SO). In addition, the internal reference (CH_2_FCN) that has a hydrogen and fluorine atom was chosen to increase the accuracy. The formula shown below was used to calculate the amount of components.

$$P_{sample}=\frac{S_{sample}}{S_{std}}\times \frac{N_{std}}{N_{sample}}\times \frac{m_{std}}{m_{sample}}\times \frac{M_{sample}}{M_{std}}\times P_{std}$$

where *S* = Integrated area of the peak, *N* = Number of protons atoms in the functional group), *m* = Weighted mass, *M* = Molecular weight, and *P* = Purity.

### 2.5. In Vitro Studies

#### 2.5.1. Cell Culturing

Human epidermoid carcinoma A431 and human lung carcinoma A549 cancer cell lines were tested. These cell lines were distributed by the European Collection of Cell Cultures (ECACC, Salisbury, UK) and purchased through Sigma Aldrich (Merck KGaA, Darmstadt, Alemania). Cells were cultured in Dulbecco’s Modified Eagle’s Medium (DMEM) “complete” (i.e., DMEM with phenol red, supplemented with Fetal Calf Serum (FCS, 10.0% *v*/*v*), Penicillin–Streptomycin (PS solution; 0.2% *v*/*v*), and GlutaMax (GM, 0.9% *v*/*v*)). Both cell lines were cultured under humidified conditions (37 °C atmosphere containing 7.0% CO_2_) in 75 cm^2^ flasks and sub-cultured (1:3–1:6) upon reaching 70–80% confluency (once per week). Media were refreshed every 3 days; cells were passaged for 4–8 weeks maximum.

#### 2.5.2. Cell-Irradiation Setup

The same cell-irradiation system was used as published previously from our group [51] that consisted of a Ditabis thermostat (980923001) fitted with two flat-bottomed microplate thermoblocks (800010600) and a 96-LED array fitted to a standard 96-well plate. The λ_exc_ = 520 nm LED with power density (10.9 mW/cm^2^) (OVL-3324), fans (40 mm, 24 V DC, 9714839), and power supply (EA-PS 2042-06B) were obtained from Farnell.

#### 2.5.3. Cytotoxicity Assay

Cells were seeded at a density of 5 × 10^3^/mL for A549 and 8 × 10^3^/mL for A431 in 96-well plates at t = 0 h using Opti-MEM complete without phenol red (100 μL) and incubated for 24 h at 37 °C, under 7% of CO_2_. Subsequently, aliquots (100 μL) of six different concentrations between 50 ng/mL and 50 µg/mL (0.7 ng/mL to 2.83 µg/mL based on metal content) of freshly prepared stock **RuBIS** CPNs suspension or complex **2** solution in Opti-MEM were added to three adjacent wells as a triplicate. A minimum amount of DMSO (<0.5%) was used to dissolve the compounds, which does not harm the cells in each well, including in the control wells. After incubation in the dark for an additional 24 h, the plates were irradiated for 60 min with green light (λ_exc_ = 520 nm, power density 10.92 mW/cm^2^, light dose = 39.3 J/cm^2^). After irradiation, the plates were incubated in the dark for an additional 48 h either in normoxia or hypoxic incubator. Then, the cells were fixed using cold (4 °C) TCA (10% *w*/*v*; 100 μL). Subsequently, TCA was removed from the wells, and the plates were washed with water (×5), stained with SRB (0.6% *w*/*v* in acetic acid (1% *v*/*v*; 100 μL) for 30 min, washed with acetic acid (1% *v*/*v*; ≈300 μL), and air dried overnight. After solubilizing the SRB dye with Tris-base (10 mm; 200 μL), the absorbance was read in each well at λ = 510 nm by using a M1000 Tecan Reader.

The fraction of viable cells in each well was calculated using SRB absorbance (Excel and GraphPad Prism software) obtained from triplicates for each concentration. The relative cell viabilities were obtained by dividing the average absorbance of the treated wells by that observed in the untreated wells for three independent biological replicates (three different passage numbers per cell line). The average cell viability was plotted versus log (concentration) (μM), including the standard deviation error of each point. The effective concentration (EC_50_) was calculated by using the dose–response curve for each cell line (dark vs. irradiated conditions), by fitting the curves to a non-linear regression function, as relative cell viability, and obtaining a variable Hill slope from Equation (1)

Y = 100/(1+10((log_10_EC_50_−X) ⋅ Hill Slope)). (1)

#### 2.5.4. Cellular Uptake Measurements

A431 cells (5 × 10^5^) were seeded in 12-well plates, incubated for 24 h under normoxic conditions, and treated with **RuBIS** CPNs (25 μg/mL) or complex **2** (19 μg/mL) for 2 h. Then, cells were washed thrice with cold (4 °C) PBS (3 × 2 mL) to remove any compound attached outside the cells. Then, the cells were trypsinized and collected into a 2 mL Eppendorf tube in Opti-MEM media. Cells were counted on a BioRad cell-counting board and carefully washed once with cold PBS to remove trypsin. Then, collected cells were centrifuged at 3000 rpm for 5 min. The resulting cell pellet was digested using 65% HNO_3_ at 100 °C overnight in a hot air oven. Once back to room temperature, the total volume was completed to 10 mL using Milli-Q® water. The ruthenium content and the standard deviation values of these solutions were analyzed on the duplicate experimental results using the Perkin Elmer NexION 2000 (PerkinElmer, Shelton, CT, USA) of an inductively coupled plasma mass spectrometer (ICP-MS, PerkinElmer, Shelton, CT, USA).

#### 2.5.5. Endocytosis Inhibition Studies

A431 cells (5 × 10^5^ cells) were seeded in 12-well plates, incubated for 24 h under normoxic conditions, and then treated with NaN_3_ (active uptake inhibitor, 15.4 mM), NH_4_Cl (20 mM), or Dynasore (dynamin-dependent endocytosis inhibitor, 80 μM) for 1 h; alternatively, the cells were incubated at 4 °C for 1 h. After that, the cells were incubated with either **RuBIS** CPNs (50 μg/mL) or complex **2** (38 μg/mL) for 3 h in the regular incubator for the inhibitor samples or at 4 °C for the low temperature samples. Then, the cells were treated as in the normal cellular uptake study.

#### 2.5.6. ICP-MS Analysis

The sample was digested in nitric acid (65%, Suprapur^®^, Merck, Darmstadt, Germany), while diluted (1%) nitric acid was used as a carrying solution. NIST-traceable 1000 mg/L elemental standards (TraceCERT^®^, Fluka Chemie GmbH, Buchs, Switzerland) were used for the calibration and as internal standards. Calibration standards were prepared in a Secuflow fume hood (SCALA, Wangen, Germany) to prevent contamination, and MiliQ^®^ was used in all sample preparation and analysis steps. The measurements were analyzed using the NexION^®^ 2000 ICP-MS (PerkinElmer, Shelton, CT, USA) equipped with a concentric glass nebulizer and Peltier-cooled glass spray chamber. An SC2 DX autosampler (PerkinElmer, Shelton, CT, USA) was used for sample introduction. Data recording and processing was done by using Syngistix^TM^ Software (v.2.5, PerkinElmer, Shelton, CT, USA). Trace elemental calibration standards were prepared at 0, 1, 4, 20, and 100 µg/L using an NIST-traceable 1000 mg/L Ru standard. An additional set of calibration for Ru (0, 0.1, 0.5, 2.5, and 10 µg/L) was prepared for samples that were anticipated to contain low-level Ru. Samples were analyzed without dilution to minimize the possibility of contamination, using 10 μg/L Rh and In as the internal standard. To check the calibration, samples were analyzed through a repeated measurement of one of the calibration standards and a blank measurement. Curves with correlation coefficient higher than 0.999 were accepted for the calibration.

#### 2.5.7. Singlet Oxygen (^1^O_2_) Production Studies

Singlet oxygen generation measurements were conducted in cell-growing medium using 9, 10-anthracenediyl-bis(methylene) dimalonic acid (ABMDMA) as an ^1^O_2_-specific probe. ABMDMA is a hydrophilic anthracene derivative that reacts with ^1^O_2_ to produce the corresponding endoperoxide [52], thereby lowering the absorbance at 400 nm. For the experiment, 0.1 mM of ABMDMA (in Opti-MEM) was mixed with **RuBIS** CPNs (25 μg/mL), which was previously dispersed in Opti-MEM cell culture media. The resulting samples were taken into a 3 mL quartz cuvette to record the absorbance in the dark or following green light irradiation (λ_exc_ = 520 nm, 39.3 J/cm^2^). Absorption spectra were recorded initially every 30 s during the first 1 min of continuous light irradiation and successively every 1 min interval during 6 min. The reference rose Bengal dye caused significant changes to the absorption spectra of ABMDMA at 400 nm, which indicated the production of ^1^O_2_ with a quantum yield of Φ_Δ_ = 0.68 [53].

## 3. Results and Discussion

### 3.1. Synthesis and Characterization

**Figure 3:**
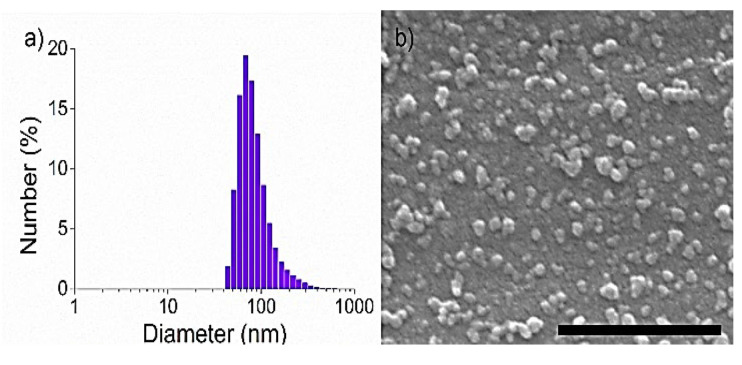
(**a**) Size distribution of **RuBIS** CPNs, measured using dynamic light scattering in PBS solution (average diameters of 50 ± 12 nm). (**b**) Representative SEM image of **RuBIS** CPNs, scale bar: 500 nm.

In a typical synthesis, complex **1** was refluxed in 1 mL of MilliQ^®^ water under N_2_ atmosphere. Subsequently, 1 mL of ethanol solution containing one equivalent of **BIS** ligand was slowly injected in the Ru complex solution, and the reflux was maintained for 1 h. Afterwards, the solution was cooled down to room temperature, and a saturated KPF_6_ solution was added to the mixture, resulting in a brown precipitate. The solid was centrifuged, washed with cold ethanol, and freeze-dried for subsequent storage and characterization. Fourier-transform infrared spectroscopy (FTIR) of the freeze-dried solid showed the presence of the typical peaks of both **BIS** (3000 cm^−1^, 1509 cm^−1^ and 1472 cm^−1^) [54] and of complex **1** (1532 cm^−1^ and 1098 cm^−1^). More importantly, new bands at 839 cm^−1^ and 429 cm^−1^ assigned to antisymmetric stretching of PF_6_ and Ru-N stretching modes, respectively, confirmed the coordination of the **BIS** ligand to complex **1** and the presence of PF_6_^−^ as a counterion (Figure S5) [55]. Dynamic light scattering (DLS) analysis of colloidal CPNs in Milli-Q^®^ water showed an average hydrodynamic diameter of 93 ± 46 nm (Figure 3a) in agreement with the value of 50 ± 12 nm of average size found using scanning electron microscopy (SEM) (Figure 3b). Moreover, the colloidal stability of the nanoparticles for at least 24 h in the dark was also corroborated by DLS analysis in a 20 mg/mL BSA-containing PBS solution, which was used as a physiological media model (Figure S6). The absence of the diffraction pattern observed from the X-ray powder diffraction (XRD) indicated the amorphous nature of as-obtained **RuBIS** CPNs (Figure S7). Finally, inductively coupled plasma mass spectrometry (ICP-MS) analysis of the nanoparticles showed 6.9 ± 0.2 wt % of Ru content (Figure S8) and ^1^H and ^19^F nuclear magnetic resonance (NMR) of dissolved CPNs in acidic solvent, using CH_2_FCN as the internal reference (see the procedure in the Materials and methods section and NMR spectra in Figure S9a,b), which allowed us to propose the chemical formula [[Ru(biqbpy)]_1.5_(bis)](PF_6_)_3_. The stoichiometric deviation from the theoretical expected ratio of the components for a linear polymer ([Ru(biqbpy)]:**BIS** = 1:1) is quite archetypal for CPNs. This is attributed to the out-of-equilibrium synthetic conditions that lead to the fast precipitation process of oligomeric species with different stoichiometry [45,46,48,49,56,57]. In any case, the Ru complex payload value of 41 wt % is more than four-fold higher than most conventional metallodrug-loaded polymer carriers known to date (typically less than 10%) [58]. It is worth mentioning that the characterization analysis was successfully performed for at least three different batches to assure the reproducibility of the synthetic methodology.

### 3.2. Photoreactivity of RuBIS CPNs

#### 3.2.1. Monitorization by UV-Vis

**Figure 4:**
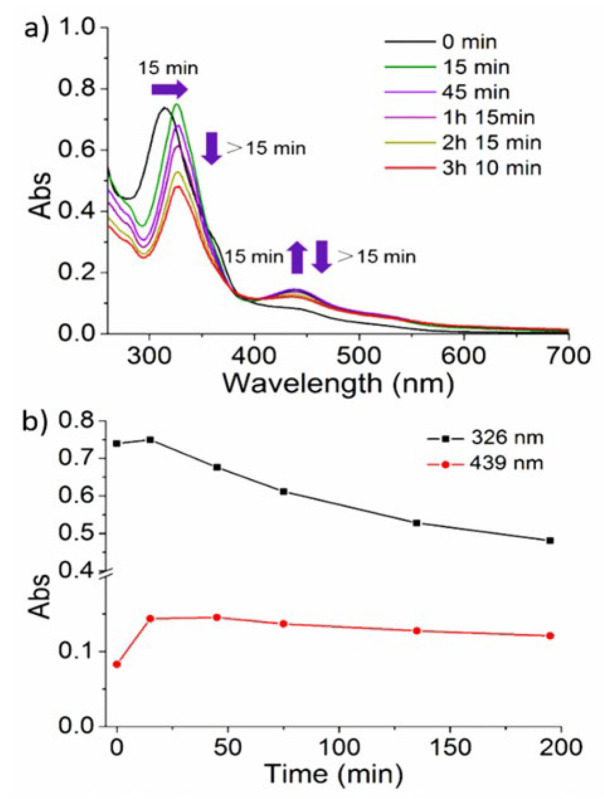
(**a**) Evolution of the UV-Vis spectra of a PBS suspension of **RuBIS** CPNs upon green light (λ_exc_ = 532 nm); (**b**) plot of absorption changes at 326 nm (black) and 439 nm (red).

UV-Vis spectroscopy of fresh-made PBS suspensions of the nanoparticles showed an absorption band with a maximum at λ_max_ = 315 nm and two shoulders at λ_abs_ = 365 nm and 439 nm lengthening up to 600 nm (Figure 4, black curve, time = 0 min). No spectral changes were observed in the dark upon time (3 h and 10 min, Figure S10) while, as expected, significant changes were found under irradiation. For these experiments, two different irradiation wavelengths were selected: (i) 450 nm, close to the CPNs absorption maximum in the visible region (Figure S11) and (ii) 532 nm, taking advantage of the tail of the broad absorption band (Figure 4). This last wavelength should be not only suitable for triggering the photosubstitution reactions but it also less harmful than blue light to living cells with also deeper penetration in biological tissues.

Irradiation with 450 nm (0.35 W/cm^2^) for the first 10 min showed a red shift of the main band, from λ_max_ = 315 to 326 nm and an intensity increase in the shoulder at λ_abs_ = 439 nm. Irradiation for longer periods (up to 2.5 h) evidenced an intensity decrease in both bands, including the shoulder at λ_abs_ = 365 nm until its disappearance (Figure S11) and an isosbestic point at λ_iso_ = 406 nm. This time-dependent two-step process was tentatively explained by an initial fast formation of photoinduced intermediate species (t_irradiation_ ≤ 10 min) and a subsequent slower photochemical reaction (t_irradiation_ > 10 min) leading to the formation of the final photoproduct. Irradiation with green light (λ_exc_ = 532 nm, 0.42 W/cm^2^) yielded similar results, although the spectral changes occurred more slowly due to the lower absorption of the **RuBIS** CPNs at the used wavelength. A red shift from λ_max_ = 315 to 326 nm and an increase in intensity at λ_abs_ = 439 nm was observed for the first 15 min of irradiation, while longer irradiation periods (up to 3 h) induced a decrease in the two bands (326 nm and 439 nm) and the formation of an isosbestic point (Figure 4). Interestingly, similar evolution in UV-Vis spectra was observed for the molecular complex **1** under both blue and green light irradiation (Figure S12) [14], which suggests a similar photo-induced process toward the active photoproduct.

#### 3.2.2. HPLC Studies

Complementary high-performance liquid chromatography (HPLC) studies were done to study the photoproducts resulting upon **RuBIS** CPNs green light irradiation. Its intrinsic selectivity and sensitivity compared to ^1^H-NMR or UV-Vis spectroscopy allows the precise quantification of photoproducts as well as the ability to differentiate final Ru-containing by-products more specifically than inductively coupled plasma mass spectrometry (ICP-MS). Before any measurement with **RuBIS** CPNs, a stock solution in PBS buffer of complex **1** (1 mg/mL), was irradiated with green light (1.1 W/cm^2^) for 20 h (Figure S13). Elution of the irradiated sample resulted in a single peak at 18.3 min assigned to the active complex [Ru(biqbpy)(H_2_O)_2_]^2+^, as confirmed by ESI mass spectrometry (Figure S14) with the corresponding *m*/*z* = 271.0 (calc. *m*/*z* = 270.8). As complex **1** to active form conversion was quantitative under such experimental conditions [14], a calibration curve of the fully activated complex was obtained at different concentrations (R^2^ = 0.996) (Figure S15).

**Figure 5:**
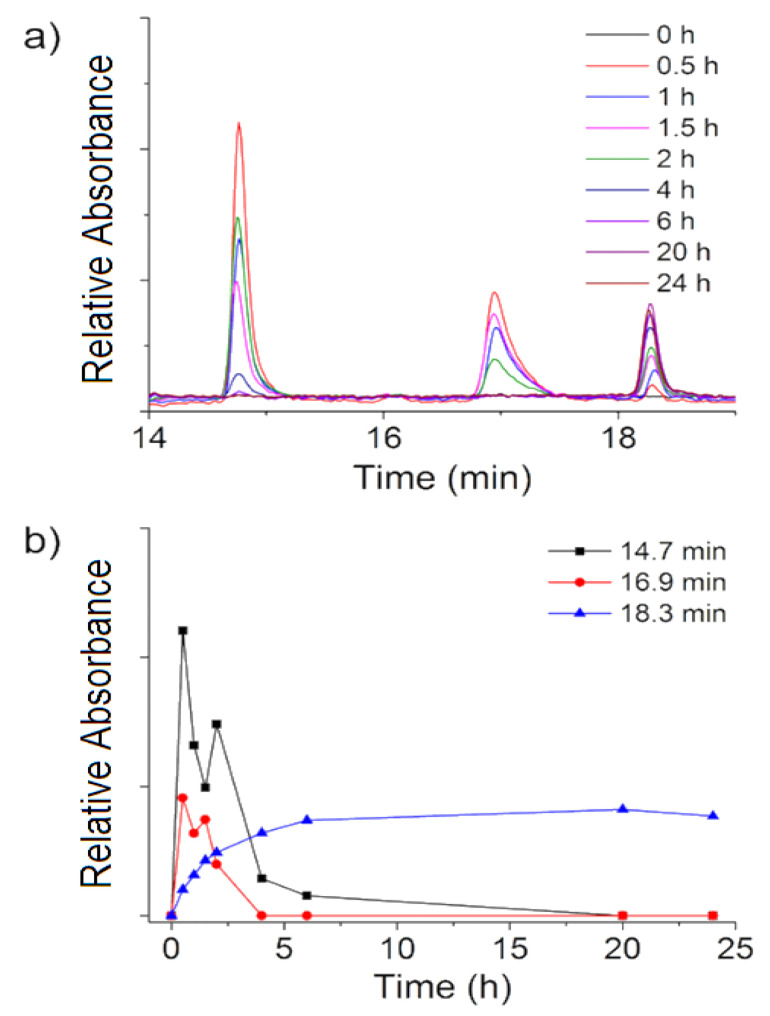
(**a**) Time-dependent HPLC chromatogram evolution under irradiation of a **RuBIS** CPNs colloidal suspension (200 µg/mL). (**b**) Plot of the time-dependent variation of relative UV absorption of each component (at given retention times) upon irradiation. Detection wavelength: λ = 305 nm.

Subsequently, a colloidal suspension of **RuBIS** CPNs in PBS buffer was irradiated (532 nm, 1.1 W/cm^2^ and 24 h), and aliquots at different irradiation times were taken, filtered, and analyzed by HPLC. To guarantee the detection of the complex released, the concentration of the initial suspension was increased with respect to that used in UV-Vis experiments up to 200 µg/mL and the irradiation time was enlarged. The results are shown in Figure 5. After the first 30 min, three different peaks with retention times of 14.7, 16.9, and 18.3 min, associated to different species, appeared after 30 min (Figure 5a and Figure S16). Further irradiation up to 6 h induced a notable increase in the peak at 18.3 min, while the two other decreased until almost complete disappearance after 20 h (Figure 5b). The formation of intermediate species at shorter irradiation times and their decrease upon prolonged irradiation to form a final product resembles the behavior observed with UV-Vis experiments (the different conversion times observed by UV-Vis vs. HPLC for the intermediates and final photoproduct were ascribed to the different CPNs concentrations used in each case). To get more detailed information on the intermediate as well as the final photoproduct chemical composition, mass spectrometry was used. Analysis of intermediate fractions at 14.7 and 16.9 min revealed oligomeric species that may come from larger fragments, such as {[Ru_2_(biqbpy)_2_bis(MeOH)_2_](PF_6_)_2_}^2+^ (found *m/z* = 828.8 calc. *m/z* = 828.7) (Figure S17), while the final photoproduct obtained at 18.3 min was identified as the target active complex [Ru(biqbpy)(H_2_O)_2_]^2+^ (Figure S14). Using the calibration curve previously obtained with this bis-aqua product, it was possible to quantitatively determine that after 24 h of green light irradiation of a 200 µg/mL **RuBIS** CPNs suspension (PBS buffer release), 1.1 µg/mL solution of the activated complex was obtained.

### 3.3. Cellular Uptake Measurements

Skin non-melanoma A431 cells were incubated in the dark for 2 h with **RuBIS** CPNs, and the amount of Ru in the cells was quantified using ICP-MS. To better define the role of the nanostructuration on the internalization and phototherapy, we repeated these experiments with a related molecular complex [Ru(biqbpy)(N-methylimidazole)_2_](PF_6_)_2_ (complex **2**), which was especially synthesized as a reference model. Complex **2**, a mononuclear analogue of complex **1** coordinated with 2 axial methylimidazole ligands, was selected as a reference molecular complex for **RuBIS** CPNs due to the analogous ruthenium coordination sphere in both systems, which facilitates and makes more suitable the comparative studies. Complex **2** was obtained upon the reaction of complex **1** with an excess of 1-methylimidazole (ratio 1:10) in EtOH under reflux and N_2_ atmosphere (for more information, see the Materials and Methods section). Remarkably, the time-dependent UV-Vis evolution of complex **2** under green light irradiation (Figure S18) is very similar to that previously observed for complex **1** (Figure S12) [14], validating its use for further biological studies.

**Figure 6:**
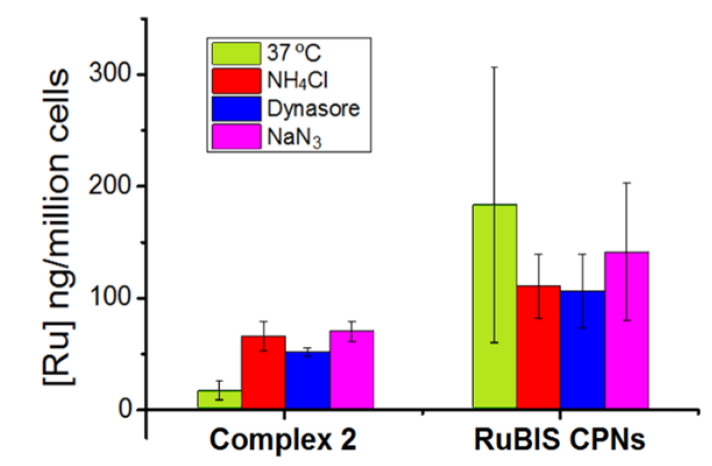
Cellular uptake quantification for Ru content in A431 cells treated with complex **2** (19 µg/mL) or **RuBIS** CPNs (25 µg/mL) nanoparticles for 2 h in the presence or absence of uptake inhibitors: ammonium chloride, dynasore, or sodium azide endocytosis.

As can be seen in Figure 6, while complex **2** uptake (16 ng Ru/million cell) is comparable to similar molecular Ru complexes previously reported [14], **RuBIS** CPNs were taken up by cells up to 11 times more (183 ng Ru/million cell). Such difference was tentatively assigned to the different internalization mechanisms. To confirm it, additional Ru quantification studies were performed co-incubating **RuBIS** CPNs or complex **2** with the endocytosis inhibitors NaN_3_, NH_4_Cl, or Dynasore, which are inhibitors of active uptake, endocytosis, and dynamin-dependent endocytosis, respectively. Indeed, cellular uptake of **RuBIS** CPNs was slightly decreased in the presence of dynasore (96.3 ng Ru/million cells), ammonium chloride (101 ng Ru/million cells), and to a lesser extent of sodium azide (138 ng Ru/million cells), compared to treatment with **RuBIS** CNPs alone (183 ng Ru/million cells). For the mononuclear complex **2**, the opposite result was observed, with a higher uptake in the presence of those inhibitors compared to treatment with 2 alone. Overall, these results suggested that endocytosis-related processes may be involved in cellular uptake of **RuBIS** CNPs, as reported previously for other nanoparticles [59], while for complex **2**, different mechanisms probably take place [60].

### 3.4. (Photo)cytotoxicity Studies

**RuBIS** CPNs dispersions (from 0.7 ng/mL to 2.83 µg/mL based on Ru content) or complex **2** solutions (from 1.0 ng/mL to 5.0 ug/mL based on Ru content) in Opti-MEM medium (with minimum amounts of DMSO < 0.5% to fully dissolve complex **2**) were seeded in human skin (A431) and lung cancer (A549) cell lines. As described in previous reports [61], the cells were seeded at t = 0, treated at t = 24 h, irradiated with green light (λ_exc_ = 520 nm, 39.3 J/cm^2^) for 1 h at 48 h, and its cell viability was quantified at t = 96 h using a standard sulforhodamine B (SRB) assay. Half-effective growth inhibition concentration (EC_50_ in µM) values at t = 96 h are shown in Table 1, and dose–response curves are shown in Figure S19. Cisplatin was also tested under the same conditions and used as control.

The EC_50_ values obtained for **RuBIS** CPNs in the dark (EC_50,dark_) of 11.88 µM and 9.10 µM decrease under irradiation (EC_50,light_) to 4.95 µM and 5.04 µM for A431 and A549 cells, respectively (Table 1). Therefore, there is a remarkable difference in the EC_50_ values for the **RuBIS** CPNs with and without irradiation, showing cell-dependent phototoxicity index (PI) values of 2.4 and 1.8 toward A431 and A549 cells, respectively. In both cases, the PI values of **RuBIS** CPNs are higher than those of the molecular complex **2** (1.7 and 1.0, respectively). From these data, the following considerations deserve to be mentioned. First, the EC_50,dark_ of **RuBIS** CPNs was higher than that of complex **2**, which was expected, as it is possibly related to the significant higher uptake. Second, the EC_50,light_ values of model complex **2** were 16.33 µM and 27.52 µM in A431 and A549 cells, respectively, which are up to 3–5 five times higher than those found for **RuBIS** CPNs, confirming the positive nanostructuration effect on the enhancement of phototoxicity. These results imply that less Ru is required to photoinduce an efficient chemotherapeutic effect, minimizing the cells death for non-irradiated cells. Moreover, the obtained EC_50,light_ values are similar to previously described monomeric complexes [14] but using almost a half-irradiation dose, which is also an additional advantage given the possible undesirable side effects that may appear from using a high irradiation doses. Last but not least, EC_50,dark_ values for **RuBIS** CPNs are three to four times higher than those of cisplatin (3.01 and 3.04 µM), i.e., less toxic; while EC_50,light_ values of **RuBIS** CPNs were close to those obtained for the photo-independent gold-standard cisplatin (EC_50_ = 3.01 µM and 3.04 µM in A431 and A549, respectively), which equate their effectiveness to drugs commercially used today. Cytotoxicity of the bis-imidazol (bis) and methylimidazole ligands are considered negligible, since a previous evaluation in our group using concentrations ranging from 0 to 100 μg/mL in different cell lines (Figure S20) corroborated their very low cytotoxic effect at the highest concentration assayed (100 μg/mL).

### 3.5. Singlet Oxygen Production

**Figure 7:**
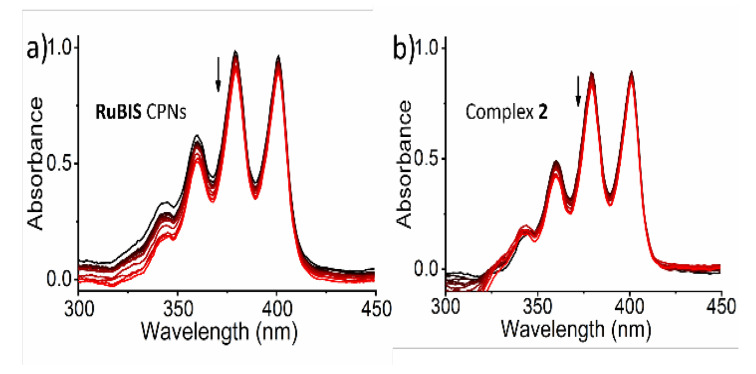
The negligible absorption spectral changes of ABMDMA upon green light irradiation in the presence of (**a**) **RuBIS** CPNs (25 μg/mL), (**b**) complex **2** (19 μg/mL). The ^1^O_2_ generation is studied in Opti-MEM medium. The arrows indicate the evolution of the spectra with time.

To discard PDT as a possible origin of the photoactivity instead of PACT, the production of singlet oxygen (^1^O_2_) upon green light irradiation of **RuBIS** CPNs was quantified. For this, the common method is to measure the near-infrared emission intensity of ^1^O_2_ (1270 nm) in CD_3_OD; though in this case and to mimic the cell culture conditions, the value of singlet oxygen quantum yield (Φ_Δ_) was indirectly determined in Opti-MEM medium using a selective water-soluble ^1^O_2_ probe (9,10-anthracenediyl-bis(methylene)-dimalonic acid, ABMDMA). In the dark, this dye absorbs light at 378 nm while in the presence of photo-generated ^1^O_2_, a less conjugated endoperoxide is formed, leading to a decrease in the absorbance at 378 nm [62]. The rose Bengal dye was used as reference, as it produces ^1^O_2_ with a known quantum yield φ_Δ_ = 0.76 [53] under green light irradiation. When **RuBIS** CPNs (25 μg/mL) was mixed with ABMDMA (100 μM) in Opti-MEM, no changes in the absorption spectra were observed with or without green light irradiation (λ_exc_ = 520 nm, see Figure 7), contrary to rose Bengal (Figure S21) [63]. The same study was performed in the same way for complex **2.** In both cases, the results excluded ^1^O_2_ production, as expected for photosubstituted active ruthenium compounds.

## 4. Conclusions

We have successfully designed and synthetized light-sensitive coordination polymer nanoparticles (CPNs) based on the polymerization of a Ru(II) polypyridyl prodrug **1** with a photocleavable bis-imidazole linking ligand **BIS**. Precise control of the reaction conditions led to the reproducible synthesis of narrow size distribution (50 ± 12 nm) CPNs with remarkable drug encapsulation yields well over those already described for other nanoencapsulation systems. The photoactivation of the **RuBIS** CPNs showed controlled release of the anticancer Ru complex [Ru(biqbpy)(H_2_O)_2_]^2+^ upon green (532 nm) irradiation, while they were stable in cell-growing medium in the dark, reducing the cell dead population and side effects in its inactivated form. Interestingly, the dose of light necessary to obtain enough cytotoxic complex from **RuBIS** CPNs in vitro (39.3 J/cm^2^) is notably lower compared to previous values published for similar green light photoactivated ruthenium systems (75 J/cm^2^) [14]. Moreover, in vitro studies demonstrated that **RuBIS** CNPs have an 11-fold increased uptake in comparison to related monomeric complexes thanks to the energy-dependent endocytosis uptake pathway triggered by the CNPs formulation. This fact determined a substantial increase in phototoxicity index in comparison with monomeric species and a light-selective cytotoxic effect close to the gold standard cisplatin. All in all, **RuBIS** CPNs demonstrates the potential of photoactivated CPNs for PACT anticancer treatments.

## Figures and Tables

**Table 1:** Cytotoxicity of **RuBIS** CPNs and complex **2** in A431 and A549 cancer cell lines in the dark and under green light (λ_exc_ = 520 nm, 39.3 J/cm^2^) irradiation.

Cell Type	Light DoseJ/cm^2^	RuBIS CPNs			Complex 2			Cisplatin		
		EC_50_ (µM)	CI [a]	PI [b]	EC_50_(µM)	CI [a]	PI [b]	EC_50_(µM)	CI [a]	PI [b]
A431	0	11.9	+0.46 −n.a.	2.4	28.1	+0.06 −0.60	1.7	3.0	+0.45 −0.41	1.1
	39.3	5.0	+0.04 −0.04		16.3	+0.55 −0.32		3.3	+0.31 −0.28	
A549	0	9.1	+0.09 −0.08	1.8	28.3	+1.16 −0.74	1.0	3.0	+0.15 −0.15	1.0
	39.3	5.0	+0.02 −0.02		27.5	+0.43 −0.37		3.0	+0.17 −0.17	

[a] Confidence interval, [b] photo indices. EC_50_ values are expressed in μM as half-maximal effective concentration (95% confidence interval are also given in μM).

## Data Availability

Data is available on the request from the corresponding author.

## Supplementary Materials

The following are available online at https://www.mdpi.com/article/10.3390/nano11113089/s1, Figure S1: ^1^H-NMR spectrum of complex 2 in (CD_3_)_2_SO, Figure S2: COSY spectrum of complex 2 in (CD_3_)_2_SO, Figure S3: ^13^C-NMR spectrum of complex 2 in (CD_3_)_2_SO, Figure S4: Mass spectrum of complex 2, Figure S5: FTIR spectra of complex 1, BIS ligand and RuBIS CPNs, Figure S6: Time-dependent DLS measurements RuBIS CPNs, Figure S7: X-ray Diffractometry of BIS ligand, KPF_6_, complex 1, and RuBIS CPNs, Figure S8: Calibration curve of the Ru for inductively coupled plasma mass spectrometry (ICP-MS), Figure S9a: ^1^H NMR spectra of RuBIS CPNs, complex 1, CH_2_FCN (internal reference) and BIS ligand, Figure S9b: ^19^F NMR spectra of RuBIS CPNs and CH_2_FCN (internal reference), Figure S10: (a) UV-vis absorption spectra of a PBS suspension of RuBIS CPNs in the dark over time. (b) Plot of absorbance at λ_abs_ = 315 and 439 nm of the RuBIS CPNs suspension in the dark. (c) DLS tracing results of the RuBIS CPNs suspension in PBS solution recorded at 0 min and 3 h 10 min, Figure S11: (a) Evolution of the UV-vis spectra of a PBS suspension of RuBIS CPNs, upon blue light irradiation (λ_ex_ = 450 nm). (b) Plot of absorbance changes at λ_abs_ = 326 nm and 439 nm, Figure S12: Evolution of the UV-vis absorbance spectra of a solution of complex 1, upon 450 nm blue or 530 nm green light irradiation under argon. Reprinted with permission from Ref. [14]. Copyright 2016 the Royal Society of Chemistry. Figure S13: The whole chromatogram of fully activated drug [Ru(biqbpy)(H_2_O)_2_]^2+^ converted from complex 1 at different concentrations, Figure S14: Mass spectrometry of the fractions at different retention times), Figure S15: The drug ([Ru(biqbpy)(H_2_O)_2_]^2+^) calibration curve, Figure S16: The whole chromatogram of the drug release of RuBIS CPNs (0–35 min), Figure S17: Mass spectra of samples with retention time, Figure S18: UV-vis absorbance spectra of a complex 2 solution after green light irradiation, Figure S19: Cytotoxicity assays with and without green light (520 nm) irradiation, Figure S20: Cytotoxicity of Bis and 1-methylimidazole free ligands, Figure S21: Absorption spectral changes of ABMDMA in the dark (a) or following green light irradiation with the light of dose 39.3 J/cm^2^, (b) in the presence of Rose Bengal (0.1 µM), Figure S22: Determination of required irradiation time for RuBIS CPNs and complex 2 activation.

## References

[1] Kladnik J., Kljun J., Burmeister H., Ott I., Romero-Canelón I., Turel I. (2019). Towards Identification of Essential Structural Elements of Organoruthenium(II)-Pyrithionato Complexes for Anticancer Activity. Chem. A Eur. J..

[2] Zhao Z., Gao P., You Y., Chen T. (2018). Cancer-Targeting Functionalization of Selenium-Containing Ruthenium Conjugate with Tumor Microenvironment-Responsive Property to Enhance Theranostic Effects. Chem. A Eur. J..

[3] Hartinger C.G., Zorbas-Seifried S., Jakupec M.A., Kynast B., Zorbas H., Keppler B.K. (2006). From bench to bedside–preclinical and early clinical development of the anticancer agent indazolium trans-[tetrachlorobis(1H-indazole)ruthenate(III)] (KP1019 or FFC14A). J. Inorg. Biochem..

[4] Gransbury G.K., Kappen P., Glover C.J., Hughes J.N., Levina A., Lay P.A., Musgrave I.F., Harris H.H. (2016). Comparison of KP1019 and NAMI-A in tumour-mimetic environments. Metallomics.

[5] Mital M., Ziora Z. (2018). Biological applications of Ru(II) polypyridyl complexes. Coord. Chem. Rev..

[6] Zeng L., Gupta P., Chen Y., Wang E., Ji L., Chao H., Chen Z.-S. (2017). The development of anticancer ruthenium(ii) complexes: From single molecule compounds to nanomaterials. Chem. Soc. Rev..

[7] Jakupec M.A., Kandioller W., Schoenhacker-Alte B., Trondl R., Berger W., Keppler B.K. (2017). Trends and Perspectives of Ruthenium Anticancer Compounds (Non-PDT). Ruthenium Complexes: Photochemical and Biomedical Applications.

[8] Smith N.A., Zhang P., Greenough S.E., Horbury M.D., Clarkson G.J., McFeely D., Habtemariam A., Salassa L., Stavros V.G., Dowson C.G. (2016). Combatting AMR: Photoactivatable ruthenium(ii)-isoniazid complex exhibits rapid selective antimycobacterial activity. Chem. Sci..

[9] Chen M., Sun W., Kretzschmann A., Butt H.-J., Wu S. (2020). Nanostructured polymer assemblies stabilize photoactivatable anticancer ruthenium complexes under physiological conditions. J. Inorg. Biochem..

[10] Sun W., Wen Y., Thiramanas R., Chen M., Han J., Gong N., Wagner M., Jiang S., Meijer M., Bonnet S. (2018). Red-Light-Controlled Release of Drug-Ru Complex Conjugates from Metallopolymer Micelles for Phototherapy in Hypoxic Tumor Environments. Adv. Funct. Mater..

[11] Imberti C., Zhang P., Huang H., Sadler P.J. (2019). New Designs for Phototherapeutic Transition Metal Complexes. Angew. Chem. Int. Ed..

[12] Lameijer L.N., Ernst D., Hopkins S.L., Meijer M.S., Askes S.H.C., Le Dévédec S.E., Bonnet S. (2017). A Red-Light-Activated Ruthenium-Caged NAMPT Inhibitor Remains Phototoxic in Hypoxic Cancer Cells. Angew. Chem. Int. Ed..

[13] Betanzos-Lara S., Salassa L., Habtemariam A., Sadler P.J. (2009). Photocontrolled nucleobase binding to an organometallic RuII arene complex. Chem. Commun..

[14] Van Rixel V.H.S., Siewert B., Hopkins S.L., Askes S.H.C., Busemann A., Siegler M.A., Bonnet S. (2016). Green light-induced apoptosis in cancer cells by a tetrapyridyl ruthenium prodrug offering two trans coordination sites. Chem. Sci..

[15] Havrylyuk D., Deshpande M., Parkin S., Glazer E.C. (2018). Ru(ii) complexes with diazine ligands: Electronic modulation of the coordinating group is key to the design of “dual action” photoactivated agents. Chem. Commun..

[16] Cuello-Garibo J.-A., Meijer M.S., Bonnet S. (2017). To cage or to be caged? The cytotoxic species in ruthenium-based photoactivated chemotherapy is not always the metal. Chem. Commun..

[17] Howerton B.S., Heidary D.K., Glazer E.C. (2012). Strained Ruthenium Complexes Are Potent Light-Activated Anticancer Agents. J. Am. Chem. Soc..

[18] Burke C.S., Byrne A., Keyes T.E. (2018). Targeting Photoinduced DNA Destruction by Ru(II) Tetraazaphenanthrene in Live Cells by Signal Peptide. J. Am. Chem. Soc..

[19] Shi G., Monro S., Hennigar R., Colpitts J., Fong J., Kasimova K., Yin H., DeCoste R., Spencer C., Chamberlain L. (2015). Ru(II) dyads derived from α-oligothiophenes: A new class of potent and versatile photosensitizers for PDT. Coord. Chem. Rev..

[20] Zhang C., Guan R., Liao X., Ouyang C., Rees T., Liu J., Chen Y., Ji L., Chao H. (2019). A mitochondria-targeting dinuclear Ir–Ru complex as a synergistic photoactivated chemotherapy and photodynamic therapy agent against cisplatin-resistant tumour cells. Chem. Commun..

[21] Farrer N.J., Salassa L., Sadler P.J. (2009). Photoactivated chemotherapy (PACT): The potential of excited-state d-block metals in medicine. Dalton Trans..

[22] Wong D.Y.Q., Ong W.W.F., Ang W.H. (2015). Induction of Immunogenic Cell Death by Chemotherapeutic Platinum Complexes. Angew. Chem. Int. Ed..

[23] Lv W., Zhang Z., Zhang K.Y., Yang H., Liu S., Xu A., Guo S., Zhao Q., Huang W. (2016). A Mitochondria-Targeted Photosensitizer Showing Improved Photodynamic Therapy Effects Under Hypoxia. Angew. Chem. Int. Ed..

[24] Lameijer L.N., Hopkins S.L., Brevé T.G., Askes S.H.C., Bonnet S. (2016). d-Versus l-Glucose Conjugation: Mitochondrial Targeting of a Light-Activated Dual-Mode-of-Action Ruthenium-Based Anticancer Prodrug. Chem. A Eur. J..

[25] Rad A.T., Chen C.-W., Aresh W., Xia Y., Lai P.-S., Nieh M.-P. (2019). Combinational Effects of Active Targeting, Shape, and Enhanced Permeability and Retention for Cancer Theranostic Nanocarriers. ACS Appl. Mater. Interfaces.

[26] Mari C., Pierroz V., Ferrari S., Gasser G. (2015). Combination of Ru(ii) complexes and light: New frontiers in cancer therapy. Chem. Sci..

[27] Barry N.P.E., Sadler P.J. (2013). Challenges for Metals in Medicine: How Nanotechnology May Help to Shape the Future. ACS Nano.

[28] Mackay F.S., Woods J.A., Heringová P., Kašpárková J., Pizarro A.M., Moggach S.A., Parsons S., Brabec V., Sadler P.J. (2007). A potent cytotoxic photoactivated platinum complex. Proc. Natl. Acad. Sci. USA.

[29] Mulcahy S.P., Li S., Korn R., Xie X., Meggers E. (2008). Solid-Phase Synthesis of Tris-heteroleptic Ruthenium(II) Complexes and Application to Acetylcholinesterase Inhibition. Inorg. Chem..

[30] Vyas N.A., Bhat S., Kumbhar A.S., Sonawane U.B., Jani V., Joshi R.R., Ramteke S., Kulkarni P., Joshi B. (2014). Ruthenium(II) polypyridyl complex as inhibitor of acetylcholinesterase and Aβ aggregation. Eur. J. Med. Chem..

[31] Alatrash N., Narh E.S., Yadav A., Kim M.-J., Janaratne T., Gabriel J., MacDonnell F.M. (2017). Synthesis, DNA Cleavage Activity, Cytotoxicity, Acetylcholinesterase Inhibition, and Acute Murine Toxicity of Redox-Active Ruthenium(II) Polypyridyl Complexes. ChemMedChem.

[32] Koch J.H., Rogers W.P., Dwyer F.P., Gyarfas E.C. (1957). The Metabolic Fate of Tris-1,10-Phenanthroline 106Ruthenium (II) Perchlorate, a Compound With Anticholinesterase and Curare-Like Activity. Aust. J. Biol. Sci..

[33] Poynton F.E., Bright S.A., Blasco S., Williams D.C., Kelly J.M., Gunnlaugsson T. (2017). The development of ruthenium(ii) polypyridyl complexes and conjugates for in vitro cellular and in vivo applications. Chem. Soc. Rev..

[34] Villemin E., Ong Y.C., Thomas C.M., Gasser G. (2019). Polymer encapsulation of ruthenium complexes for biological and medicinal applications. Nat. Rev. Chem..

[35] Karges J., Li J., Zeng L., Chao H., Gasser G. (2020). Polymeric Encapsulation of a Ruthenium Polypyridine Complex for Tumor Targeted One- and Two-Photon Photodynamic Therapy. ACS Appl. Mater. Interfaces.

[36] Sun W., Zeng X., Wu S. (2017). Photoresponsive ruthenium-containing polymers: Potential polymeric metallodrugs for anticancer phototherapy. Dalton Trans..

[37] Sun W., Parowatkin M., Steffen W., Butt H.-J., Mailänder V., Wu S. (2015). Ruthenium-Containing Block Copolymer Assemblies: Red-Light-Responsive Metallopolymers with Tunable Nanostructures for Enhanced Cellular Uptake and Anticancer Phototherapy. Adv. Healthc. Mater..

[38] Sun W., Li S., Haupler B., Liu J., Jin S., Steffen W., Schubert U.S., Butt H.J., Liang X.J., Wu S. (2017). An Amphiphilic Ruthenium Polymetallodrug for Combined Photodynamic Therapy and Photochemotherapy In Vivo. Adv. Mater..

[39] Zhang C., Guo X., Da X., Yao Y., Xiao H., Wang X., Zhou Q. (2021). UCNP@BSA@Ru nanoparticles with tumor-specific and NIR-triggered efficient PACT activity in vivo. Dalton Trans..

[40] Meijer M.S., Natile M.M., Bonnet S. (2020). 796 nm Activation of a Photocleavable Ruthenium(II) Complex Conjugated to an Upconverting Nanoparticle through Two Phosphonate Groups. Inorg. Chem..

[41] Chen Y., Jiang G., Zhou Q., Zhang Y., Li K., Zheng Y., Zhang B., Wang X. (2016). An upconversion nanoparticle/Ru(ii) polypyridyl complex assembly for NIR-activated release of a DNA covalent-binding agent. RSC Adv..

[42] Ruggiero E., Habtemariam A., Yate L., Mareque-Rivas J.C., Salassa L. (2013). Near infrared photolysis of a Ru polypyridyl complex by upconverting nanoparticles. Chem. Commun..

[43] Soliman N., McKenzie L.K., Karges J., Bertrand E., Tharaud M., Jakubaszek M., Guérineau V., Goud B., Hollenstein M., Gasser G. (2020). Ruthenium-Initiated polymerization of lactide: A route to remarkable cellular uptake for photodynamic therapy of cancer. Chem. Sci..

[44] Suárez-García S., Solórzano R., Alibés R., Busqué F., Novio F., Ruiz-Molina D. (2021). Antitumour activity of coordination polymer nanoparticles. Coord. Chem. Rev..

[45] Adarsh N., Frias C., Lohidakshan T.P., Lorenzo J., Novio F., Garcia-Pardo J., Ruiz-Molina D. (2018). Pt(IV)-based nanoscale coordination polymers: Antitumor activity, cellular uptake and interactions with nuclear DNA. Chem. Eng. J..

[46] Novio F., Lorenzo J., Nador F., Wnuk K., Ruiz-Molina D. (2014). Carboxyl Group (-CO2H) Functionalized Coordination Polymer Nanoparticles as Efficient Platforms for Drug Delivery. Chem. A Eur. J..

[47] Imaz I., Rubio-Martínez M., García-Fernández L., García F., Ruiz-Molina D., Hernando J., Puntes V., Maspoch D. (2010). Coordination polymer particles as potential drug delivery systems. Chem. Commun..

[48] Borges M., Yu S., Laromaine A., Roig A., Suárez-García S., Lorenzo J., Ruiz-Molina D., Novio F. (2015). Dual T1/T2 MRI contrast agent based on hybrid SPION@coordination polymer nanoparticles. RSC Adv..

[49] Nador F., Wnuk K., Garcia-Pardo J., Lorenzo J., Solorzano R., Ruiz-Molina D., Novio F. (2017). Dual-Fluorescent Nanoscale Coordination Polymers via a Mixed-Ligand Synthetic Strategy and Their Use for Multichannel Imaging. ChemNanoMat.

[50] Lee S., Lee J.H., Kim J.C., Lee S., Kwak S.K., Choe W. (2018). Porous Zr6L3 Metallocage with Synergetic Binding Centers for CO_2_. ACS Appl. Mater. Interfaces.

[51] Hopkins S.L., Siewert B., Askes S.H.C., Veldhuizen P., Zwier R., Heger M., Bonnet S. (2016). An in vitro cell irradiation protocol for testing photopharmaceuticals and the effect of blue, green, and red light on human cancer cell lines. Photochem. Photobiol. Sci..

[52] Chen Z.-A., Kuthati Y., Kankala R.K., Chang Y.-C., Liu C.-L., Weng C.-F., Mou C.-Y., Lee C.-H. (2015). Encapsulation of palladium porphyrin photosensitizer in layered metal oxide nanoparticles for photodynamic therapy against skin melanoma. Sci. Technol. Adv. Mater..

[53] Lutkus L.V., Rickenbach S., McCormick T.M. (2019). Singlet oxygen quantum yields determined by oxygen consumption. J. Photochem. Photobiol. A Chem..

[54] Barsukova M., Goncharova T., Samsonenko D., Dybtsev D., Potapov A. (2016). Synthesis, Crystal Structure, and Luminescent Properties of New Zinc(II) and Cadmium(II) Metal-Organic Frameworks Based on Flexible Bis(imidazol-1-yl)alkane Ligands. Crystals.

[55] Elsayed S., Jean-Claude B.J., Butler I.S., Mostafa S.I. (2012). Synthesis, structural characterization and anticancer activity of some new complexes of 6-amino-4-hydroxy-2-thiopyrimidine. J. Mol. Struct..

[56] García-Pardo J., Novio F., Nador F., Cavaliere I., Suárez-García S., Lope-Piedrafita S., Candiota A.P., Romero-Gimenez J., Rodríguez-Galván B., Bové J. (2021). Bioinspired Theranostic Coordination Polymer Nanoparticles for Intranasal Dopamine Replacement in Parkinson’s Disease. ACS Nano.

[57] Solórzano R., Tort O., García-Pardo J., Escribà T., Lorenzo J., Arnedo M., Ruiz-Molina D., Alibés R., Busqué F., Novio F. (2018). Versatile iron-catechol-based nanoscale coordination polymers with antiretroviral ligand functionalization and their use as efficient carriers in HIV/AIDS therapy. Biomater. Sci..

[58] Aryal S., Hu C.-M.J., Zhang L. (2009). Polymer—Cisplatin Conjugate Nanoparticles for Acid-Responsive Drug Delivery. ACS Nano.

[59] Manzanares D., Ceña V. (2020). Endocytosis: The Nanoparticle and Submicron Nanocompounds Gateway into the Cell. Pharmaceutics.

[60] Li Z., Zhang Y., Zhu D., Li S., Yu X., Zhao Y., Ouyang X., Xie Z., Li L. (2017). Transporting carriers for intracellular targeting delivery via non-endocytic uptake pathways. Drug Deliv..

[61] Vichai V., Kirtikara K. (2006). Sulforhodamine B colorimetric assay for cytotoxicity screening. Nat. Protoc..

[62] Yu G., Zhang M., Saha M.L., Mao Z., Chen J., Yao Y., Zhou Z., Liu Y., Gao C., Huang F. (2017). Antitumor Activity of a Unique Polymer That Incorporates a Fluorescent Self-Assembled Metallacycle. J. Am. Chem. Soc..

[63] Peterson J.C., Arrieta E., Ruggeri M., Silgado J.D., Mintz K.J., Weisson E.H., Leblanc R.M., Kochevar I., Manns F., Parel J.-M. (2020). Detection of singlet oxygen luminescence for experimental corneal rose bengal photodynamic antimicrobial therapy. Biomed. Opt. Express.

