# Supplementary material for: Photoactivable Ruthenium-Based Coordination Polymer Nanoparticles for Light-Induced Chemotherapy
Source: Nanomaterials (Basel). 2021 Nov 16;11(11):3089. doi: 10.3390/nano11113089 (PMC8617783; doi:10.3390/nano11113089)
Supplement: Supplementary file 1 [file nanomaterials-11-03089-s001.zip › nanomaterials-1440439-supplementary.pdf]

Photoactivable Ruthenium-Based Coordination Polymer Nanoparticles for Light-Induced Chemotherapy

Junda Zhang [1], Vadde Ramu [2], Xue-Quan Zhou [2], Carolina Frias [1], Daniel Ruiz-Molina [1], Sylvestre Bonnet [2,*], Claudio Roscini [1,*] and Fernando Novio [1,*]

[1] Catalan Institute of Nanoscience and Nanotechnology (ICN2), CSIC and BIST, Campus UAB, Bellaterra, 08193 Barcelona, Spain; junda.zhang@icn2.cat (J.Z.); caarolina.frias@gmail.com (C.F.); dani.ruiz@icn2.cat (D.R.-M.)

[2] Leiden Institute of Chemistry, Universiteit Leiden, Einsteinweg 55, 2333 CC Leiden, The Netherlands; ramuvadde1@gmail.com (V.R.); x.zhou@lic.leidenuniv.nl (X.-Q.Z.)

* Correspondence: bonnet@chem.leidenuniv.nl (S.B.); claudio.roscini@icn2.cat (C.R.); fernando.novio@icn2.cat (F.N.)

Table of Contents

1. Characterization of the photoactivable materials S1

2. Photoirradiation studies S7

3. *In vitro* studies S11

1. Characterization of the photoactivable materials

1.1 ¹H and ¹³C-NMR spectrum of complex 2

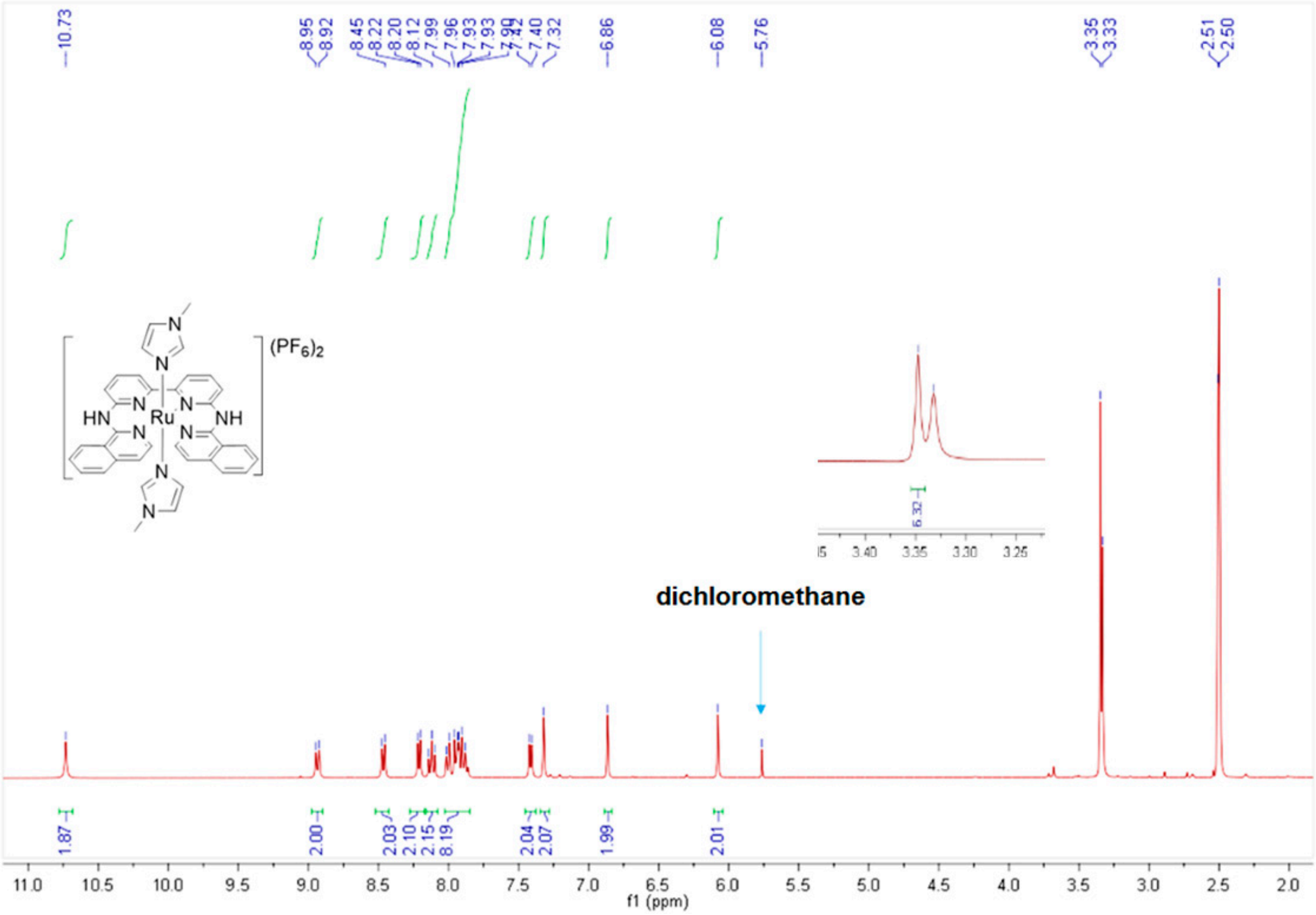

Figure S1. ¹H-NMR spectrum of complex **2** in $(CD_3)_2SO$.

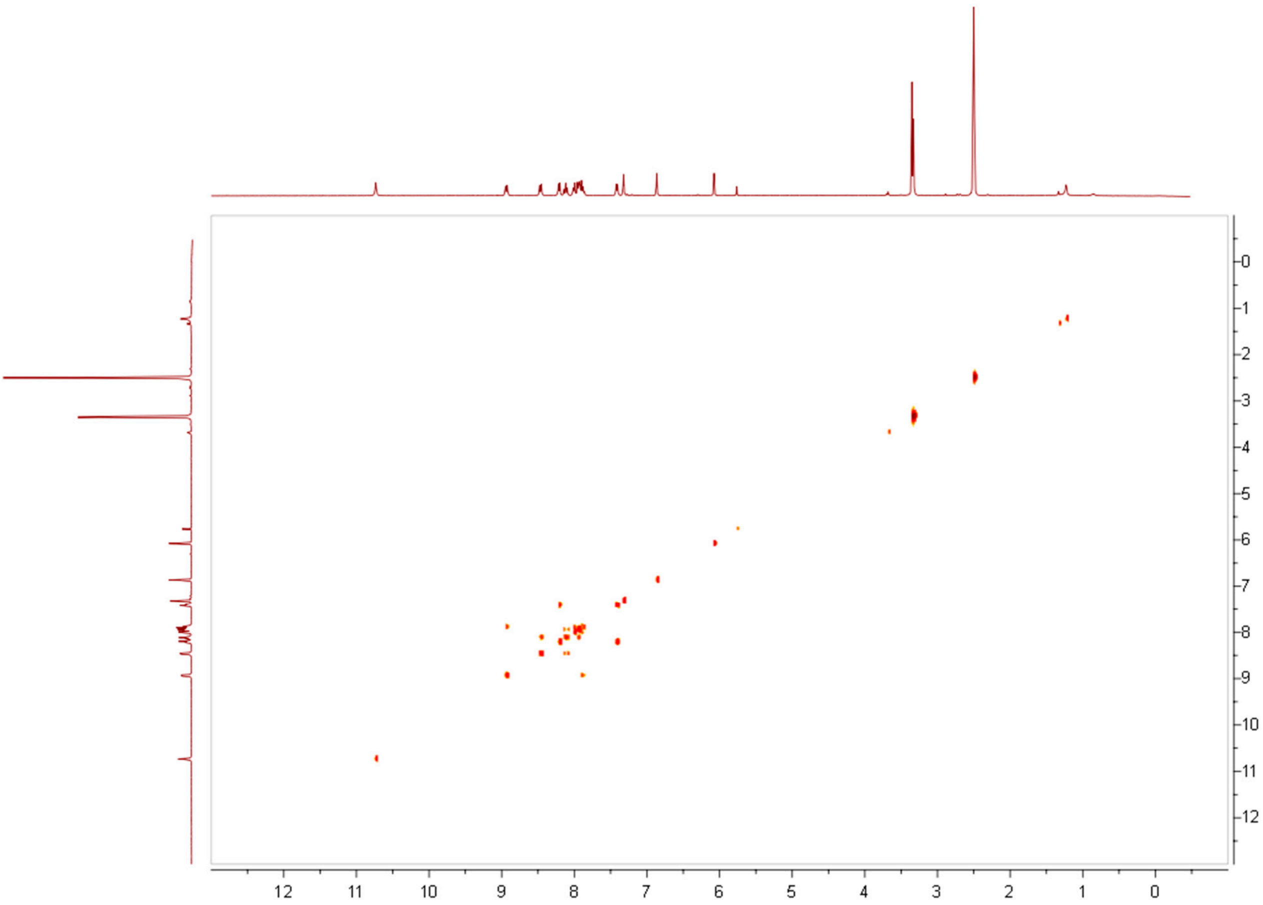

Figure S2. COSY spectrum of complex **2** in $(CD_3)_2SO$.

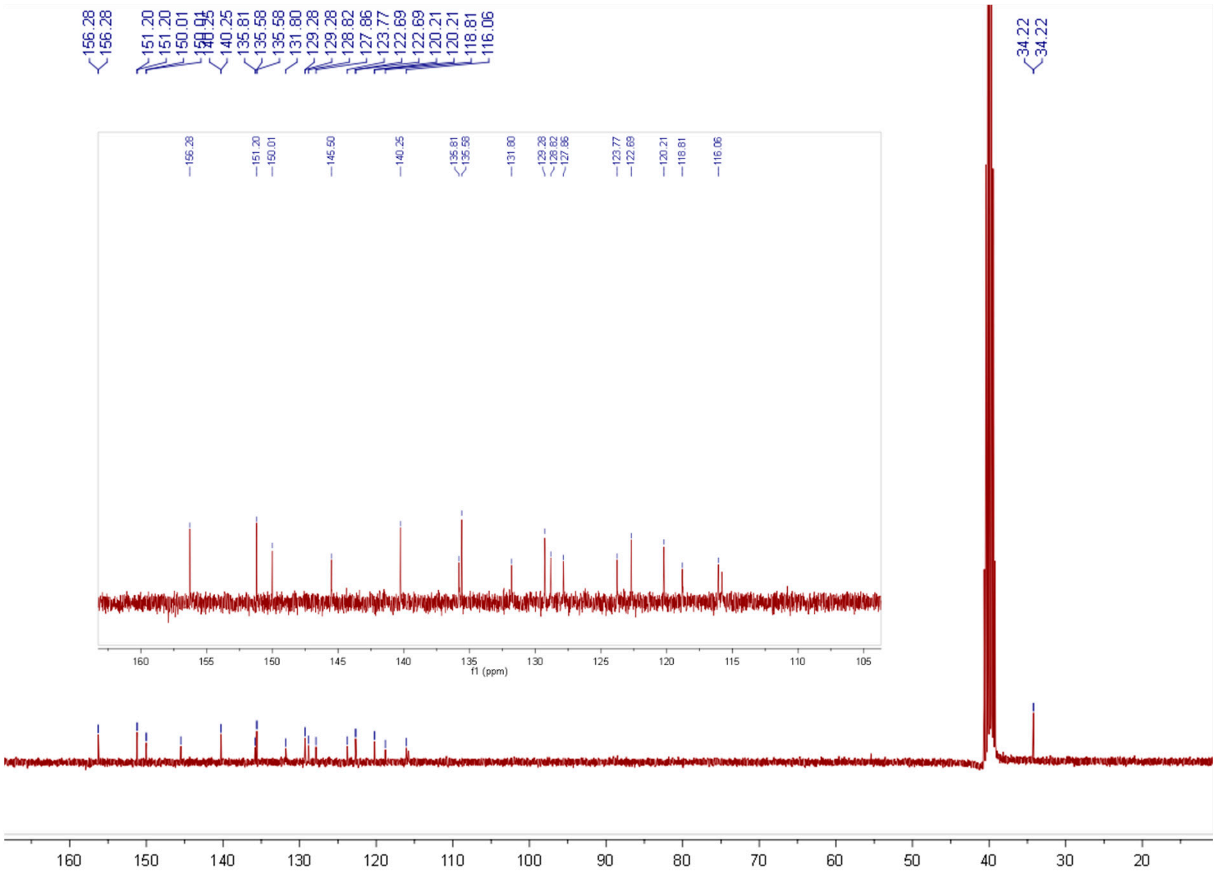

Figure S3. ^{13}C-NMR spectrum of complex **2** in $(CD_3)_2SO$.

1.2 Mass spectrum of complex 2

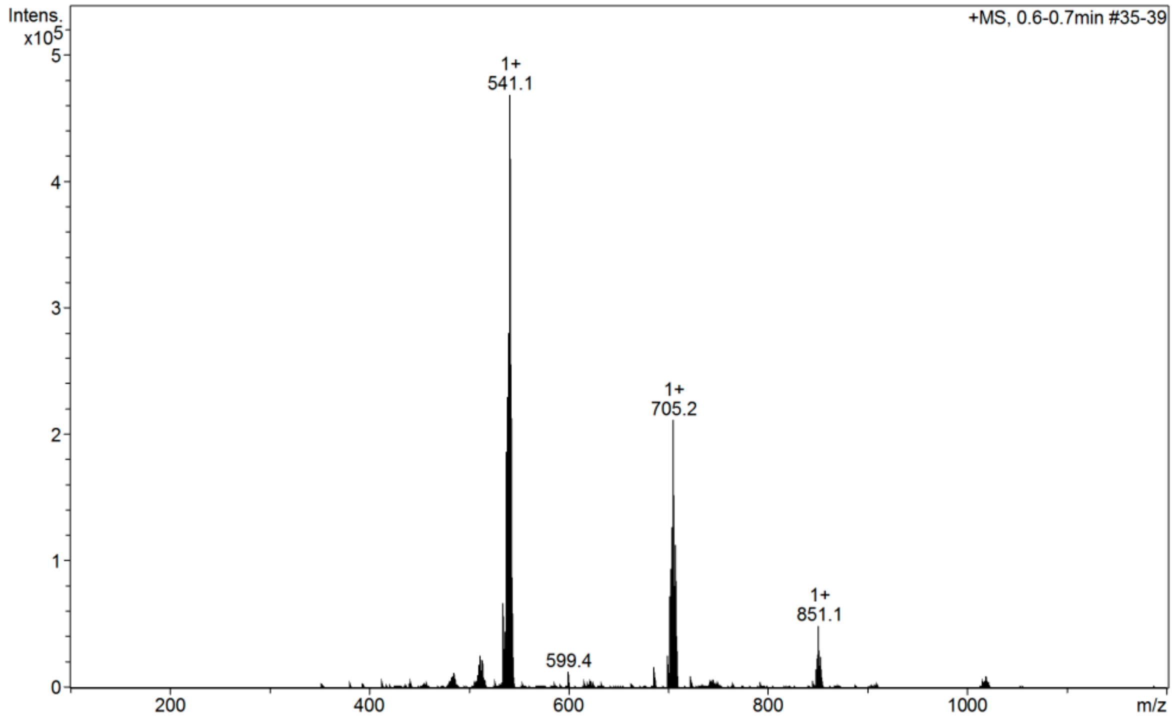

Figure S4. Mass spectrum of complex **2.**

1.3 Fourier-transform infrared spectroscopy (FTIR) spectra

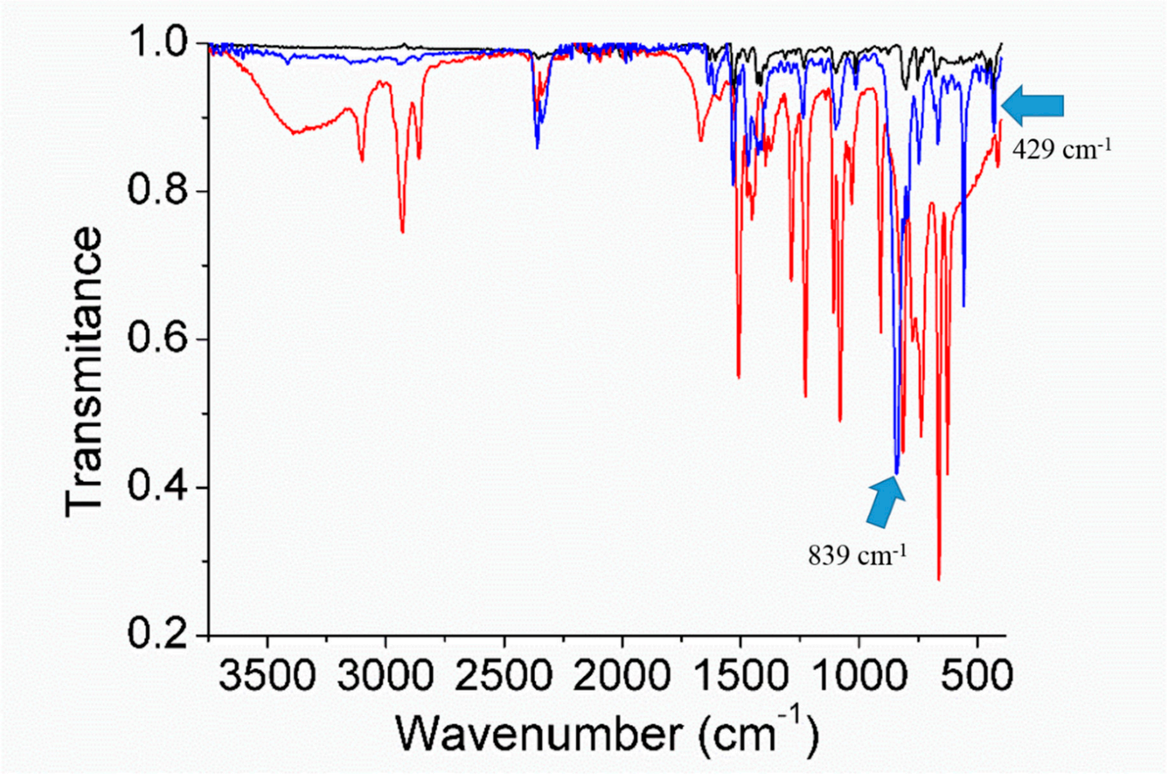

Figure S5. FTIR spectra of **1** (black), **BIS** ligand (red) and **RuBIS** CPNs (blue).

1.4 Dynamic Light Scattering (DLS)

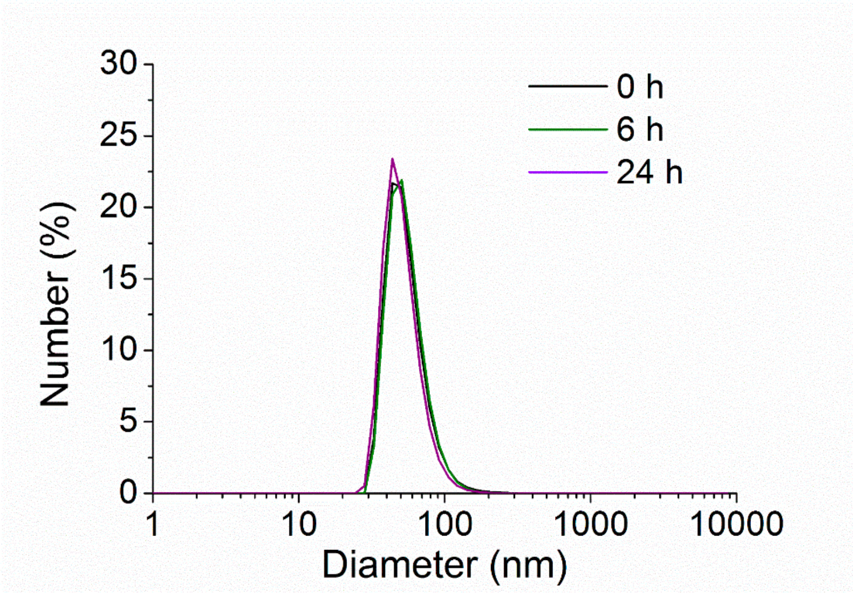

Figure S6. Time-dependent DLS measurements for a 50 ug/mL **RuBIS CPNs** solution in PBS buffer containing 20 mg/mL BSA.

1.5 Powder X-ray Diffraction (XRD)

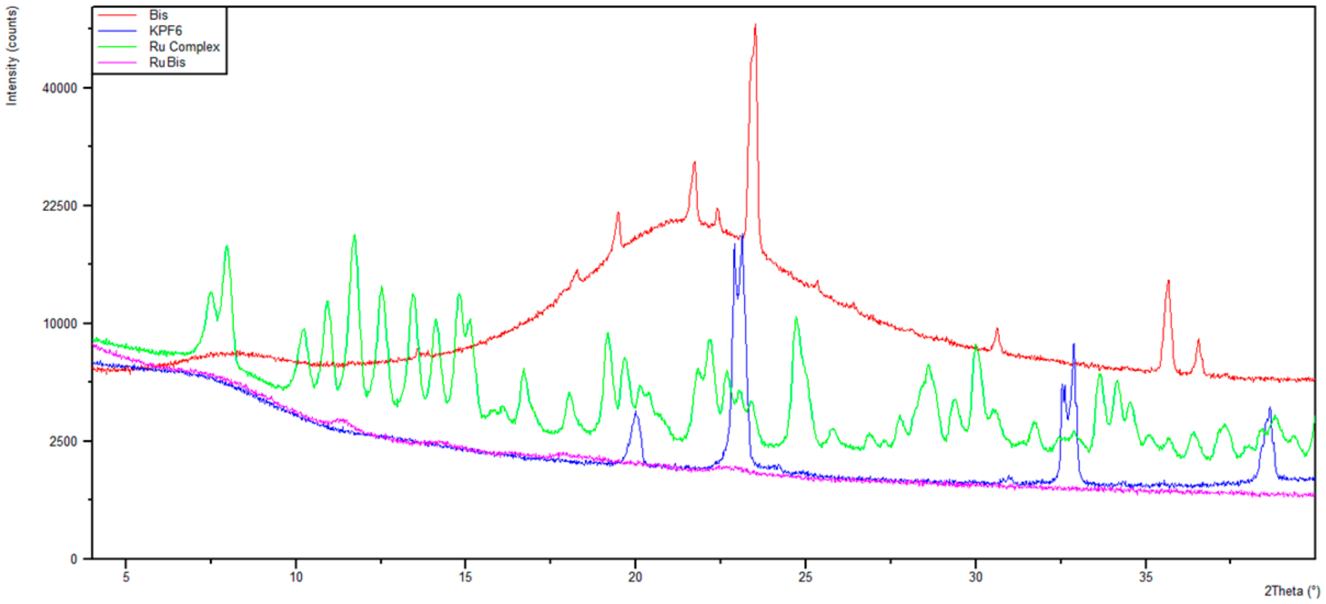

Figure S7. X-Ray Diffractometry of **BIS** ligand (red), KPF_6 (blue), **1** (green), and **RuBIS** CPNs (pink).

1.6 Inductively coupled plasma (ICP) calibration curve

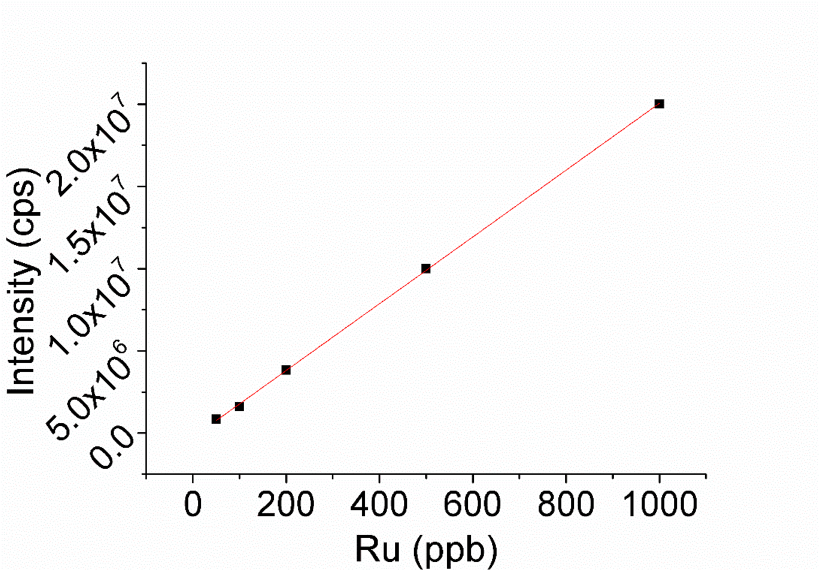

Figure S8. Inductively coupled plasma mass spectrometry (ICP-MS): the calibration curve of the Ru. From this curve and the signal intensity related to the Ru in the CPNs allowed to determine a percentage of Ru in the CPNs of 6.9 ± 0.2 *wt*%.

1.7 ^{1}H NMR and ^{19}F NMR spectra of RuBIS CPNs

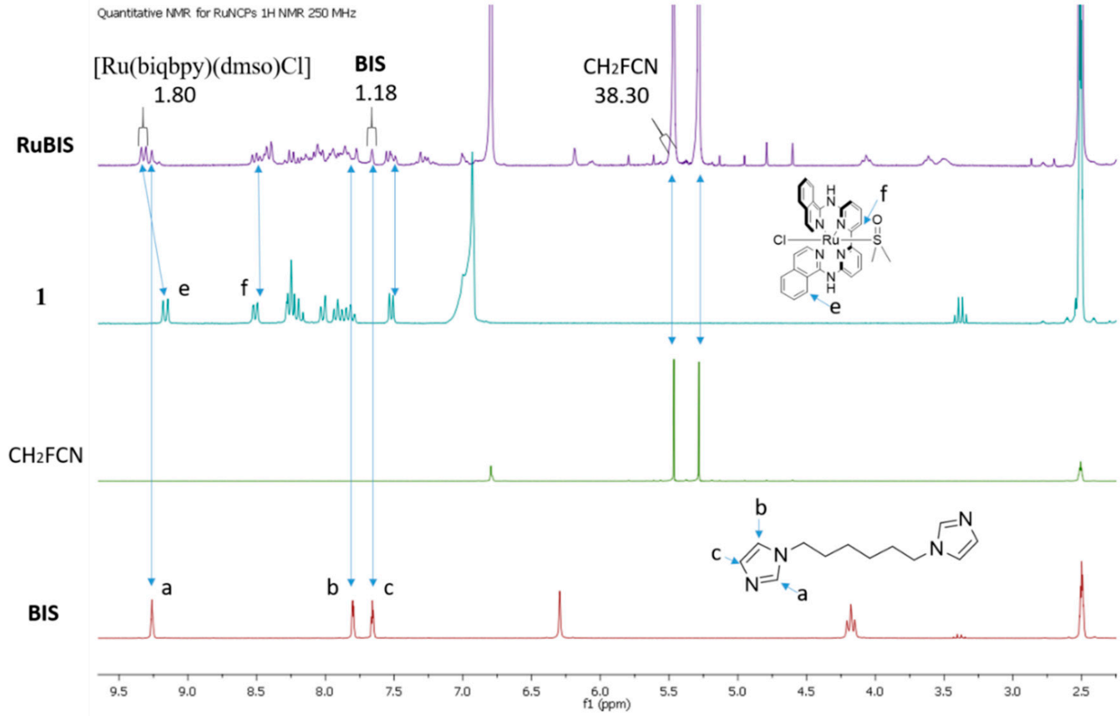

Figure S9a. ^{1}H NMR spectra of (top to bottom) **RuBIS** CPNs, complex **1**, CH_2FCN (internal reference) and **BIS** ligand. **RuBIS** CPNs were completely dissolved in deuterated dimethyl sulfoxide (DMSO-d_6) containing a small portion of deuterated hydrochloric acid (DCl); 50 µL DCl / mL DMSO-d_6.

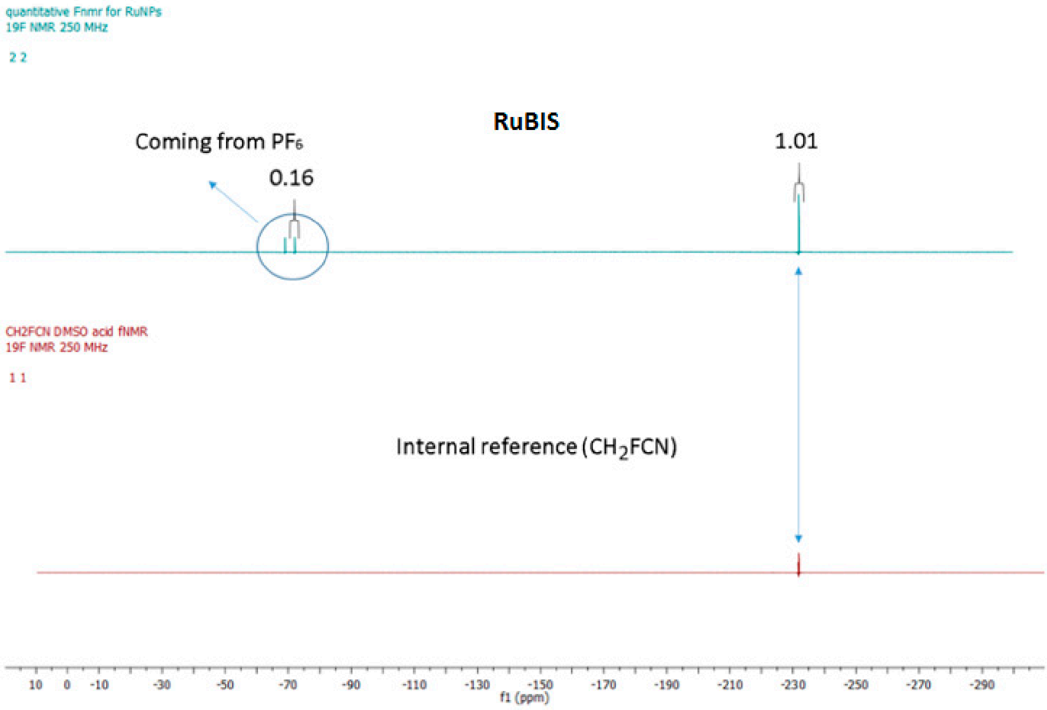

Figure S9b. ^{19}F NMR spectra of **RuBIS** CPNs and CH_2FCN (internal reference). **RuBIS** CPNs were completely dissolved in deuterated dimethyl sulfoxide (DMSO-d_6) containing a small portion of deuterated hydrochloric acid (DCl); 50 µL DCl / mL DMSO-d_6.

1.8 Stability study of RuBIS CPNs in darkness

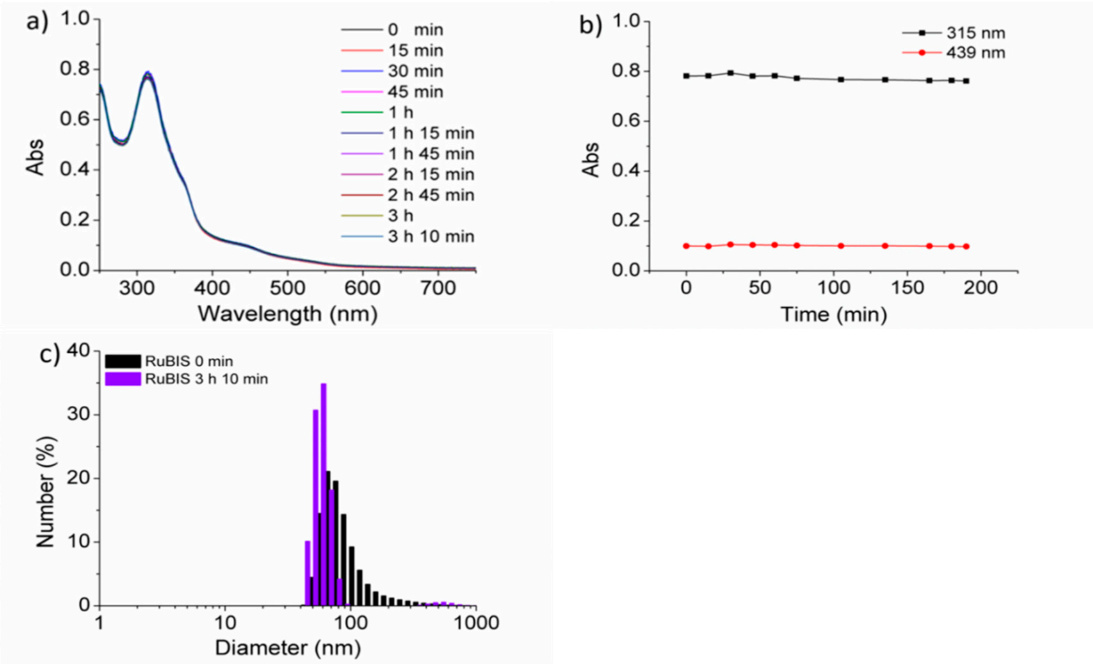

Figure S10. a) UV-vis absorption spectra of a PBS suspension of **RuBIS** CPNs in the dark over time. b) Plot of absorbance at λ_{abs} = 315 and 439 nm of the **RuBIS** CPNs suspension in the dark. c) DLS tracing results of the **RuBIS** CPNs suspension in PBS solution recorded at 0 min and 3 h 10 min.

2. Photoirradiation study

2.1 UV-vis spectra (Blue light; 450 nm)

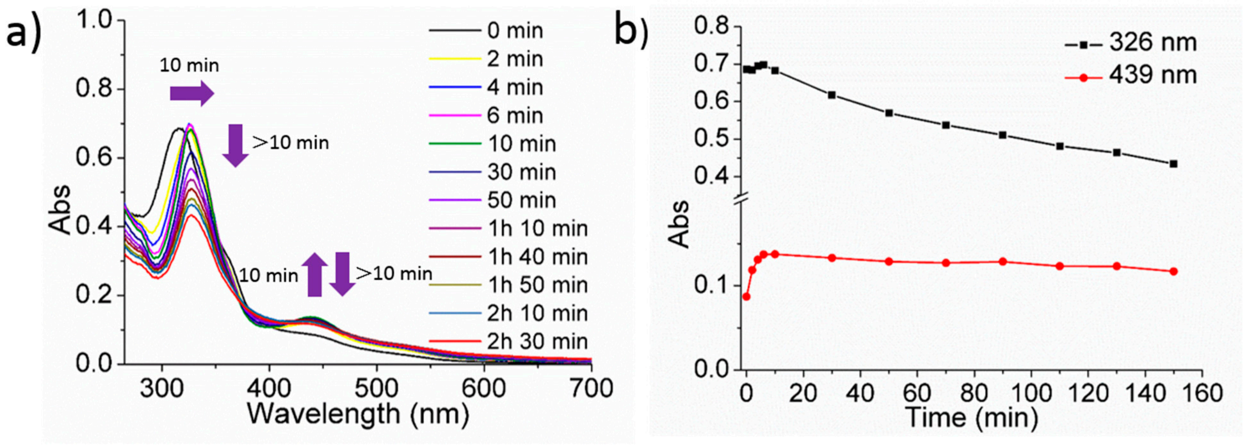

Figure S11. a) Evolution of the UV-vis spectra of a PBS suspension of **RuBIS** CPNs, upon blue light irradiation (λ_{ex} = 450 nm). b) Plot of absorbance changes at λ_{abs} = 326 nm (black) and 439 nm (red).

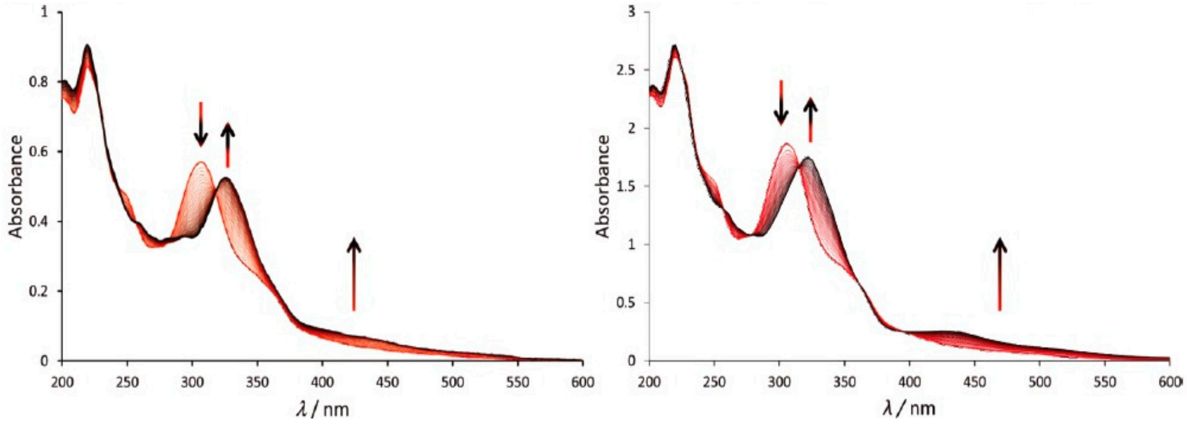

Figure S12. Evolution of the UV-vis absorbance spectra of a solution of complex **1,** upon 450 nm blue (left) or 530 nm green light (right) irradiation under argon. Reproduced from Ref. [1] with permission of the copyright holder.

2.2 HPLC drug release quantification

- Elution of the irradiated sample of complex 1 at different concentrations

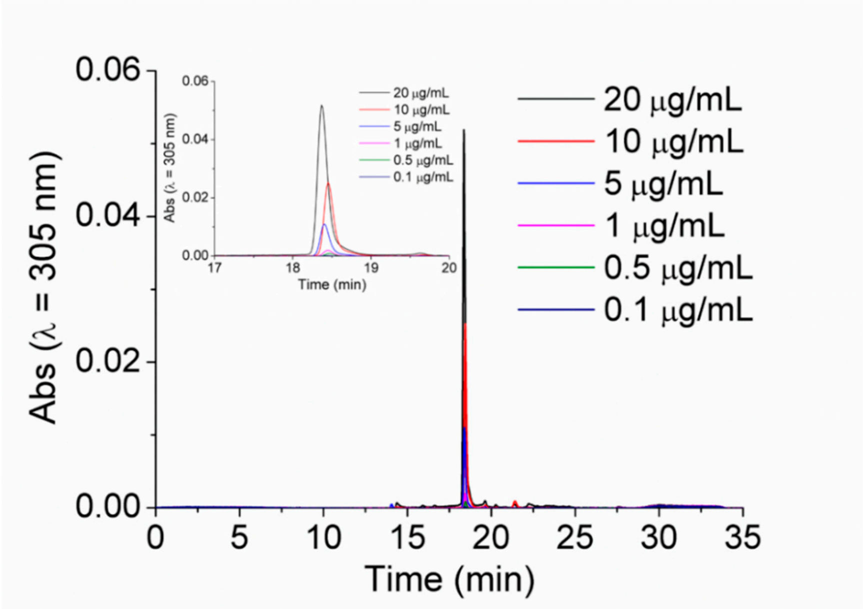

Figure S13. The whole chromatogram of fully activated drug [Ru(biqbpy)(H_2O)$_2$]$^{2+}$ converted from complex **1** at different concentrations. Experimental conditions: 1 mg/mL of complex **1** ([Ru(biqbpy)(dmso)Cl]Cl) in PBS solution under stirring was irradiated with green light (100 mW, 1.1 mW/cm^2) for 20 h to make sure that Ru complex was fully converted to the drug through photocleavage and photosubstitution process. Due to the nearly 100 % conversion of complex 1 into [Ru(biqbpy)(H_2O)$_2$]$^{2+}$, the fully converted drug solution could be used as a stock solution for obtaining the calibration curve.

- **Mass spectrometry of resulting photoproduct [Ru(biqbpy)(H_2O)$_2$]$^{2+}$**

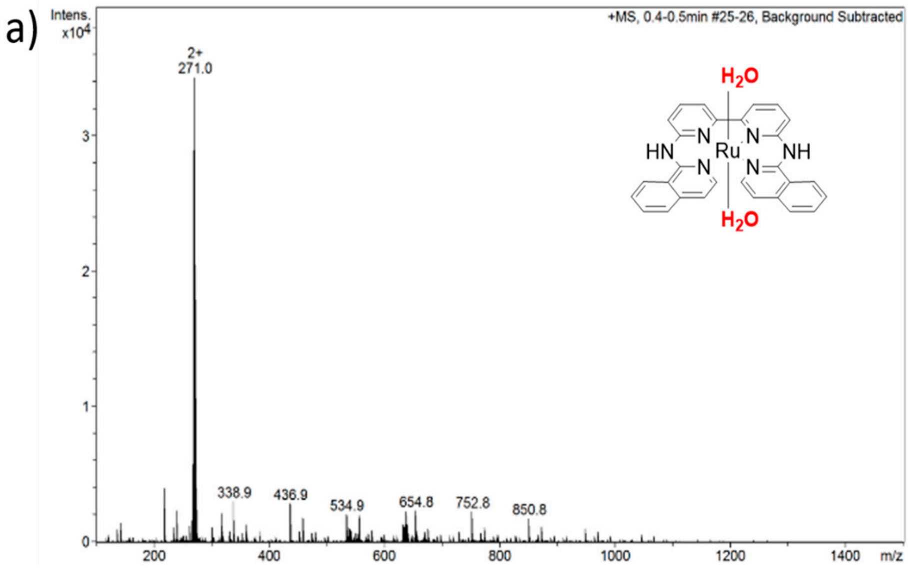

Figure S14. a) Mass spectrometry of the fractions: retention times = 18.3 min, m/z ([Ru(biqbpy)]$^{2+}$) = 271.0 (calc. m/z = 270.8.

- **$[Ru(biqbpy)(H_2O)_2]^{2+}$) calibration curve**

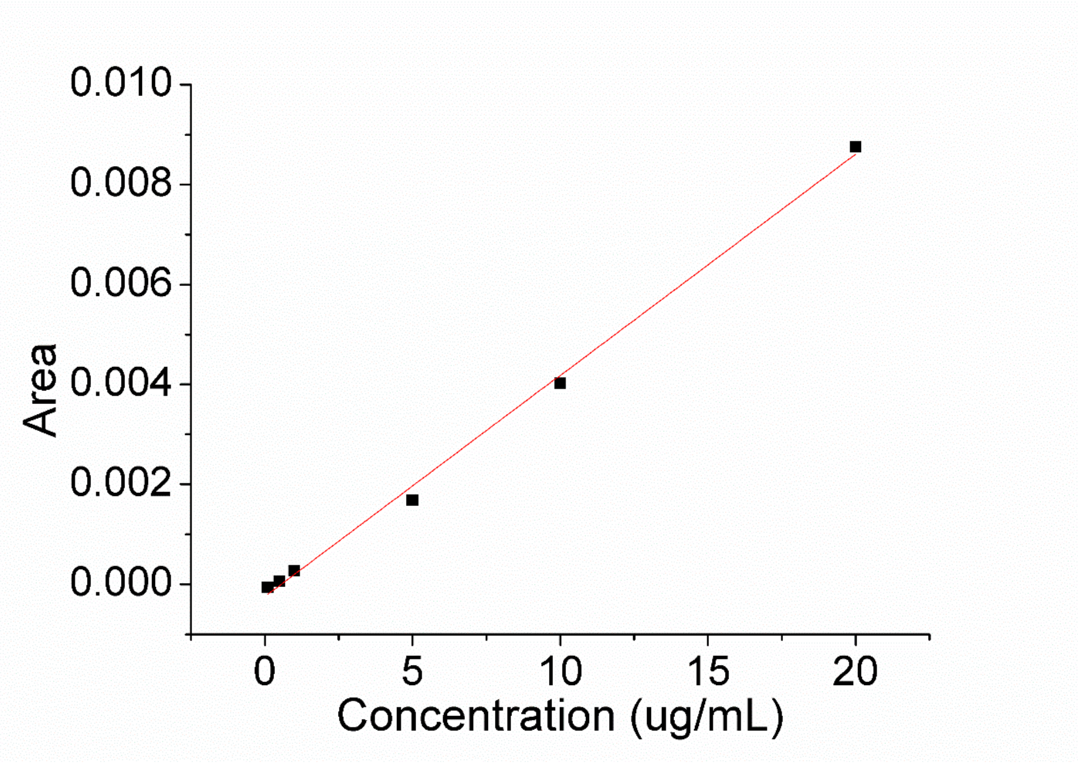

Figure S15. The drug ($[Ru(biqbpy)(H_2O)_2]^{2+}$) calibration curve using six different concentration solutions were prepared (0.1, 0.5, 1, 5, 10 and 20 μg/mL).

- **HPLC chromatogram of RuBIS drug release at different time**

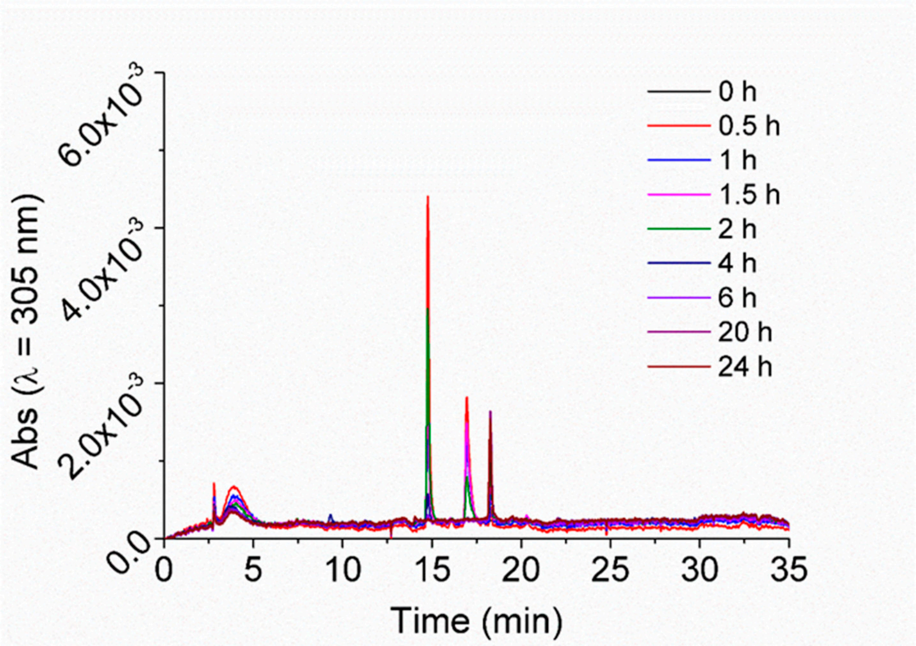

Figure S16. The whole chromatogram of the drug release of **RuBIS** CPNs (0 – 35 min).

- **Mass spectra of samples with retention time = 14.7 min**

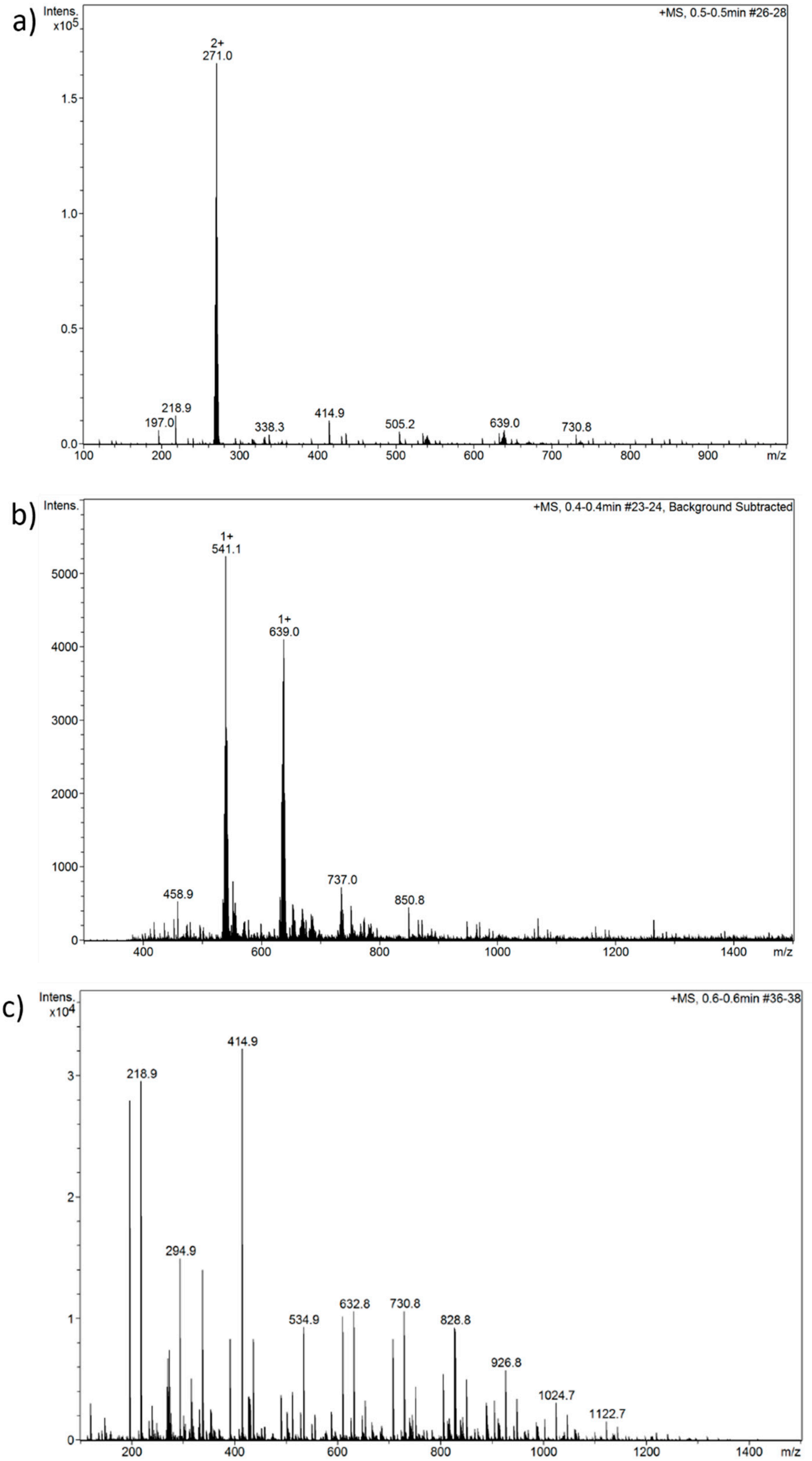

Figure S17. a,b) mass spectra of samples with retention time = 14.7 min give peaks at m/z = 271.0, 541.1, and 828.8 corresponding to $[Ru(biqbpy)]^{2+}$ (calc. m/z = 270.8), $[Ru(biqbpy)]^{1+}$ (calc. m/z = 541.0), and $\{[Ru_2(biqbpy)_2bis(MeOH)_2](PF_6)_2\}^{2+}$ (calc. *m/z* = 828.7) respectively; c) mass spectra of sample with retention time = 16.9 min, $^{2+}$ with m/z =828.8 (calc. m/z =828.7). * The presence of MeOH in $\{[Ru_2(biqbpy)_2bis(MeOH)_2](PF_6)_2\}^{2+}$ specie come from MeOH solvent used for sample dissolution.

2.3 UV-vis absorbance spectra of a complex 2 solution after green light irradiation

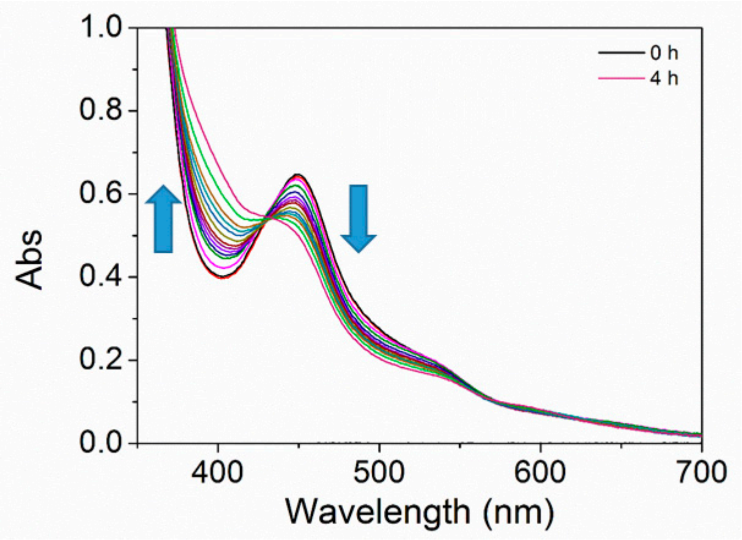

Figure S18. Evolution of the UV-vis absorbance spectra of a solution of complex **2,** upon green light (λ_{exc} = 532 nm, 0.42 W/cm^2) irradiation under argon.

3. *In vitro* studies and $^1O_2$ generation studies for RuBIS CPNs and 2 under normoxic conditions

3.1 Cytotoxicity assays with and without green light (520 nm) irradiation

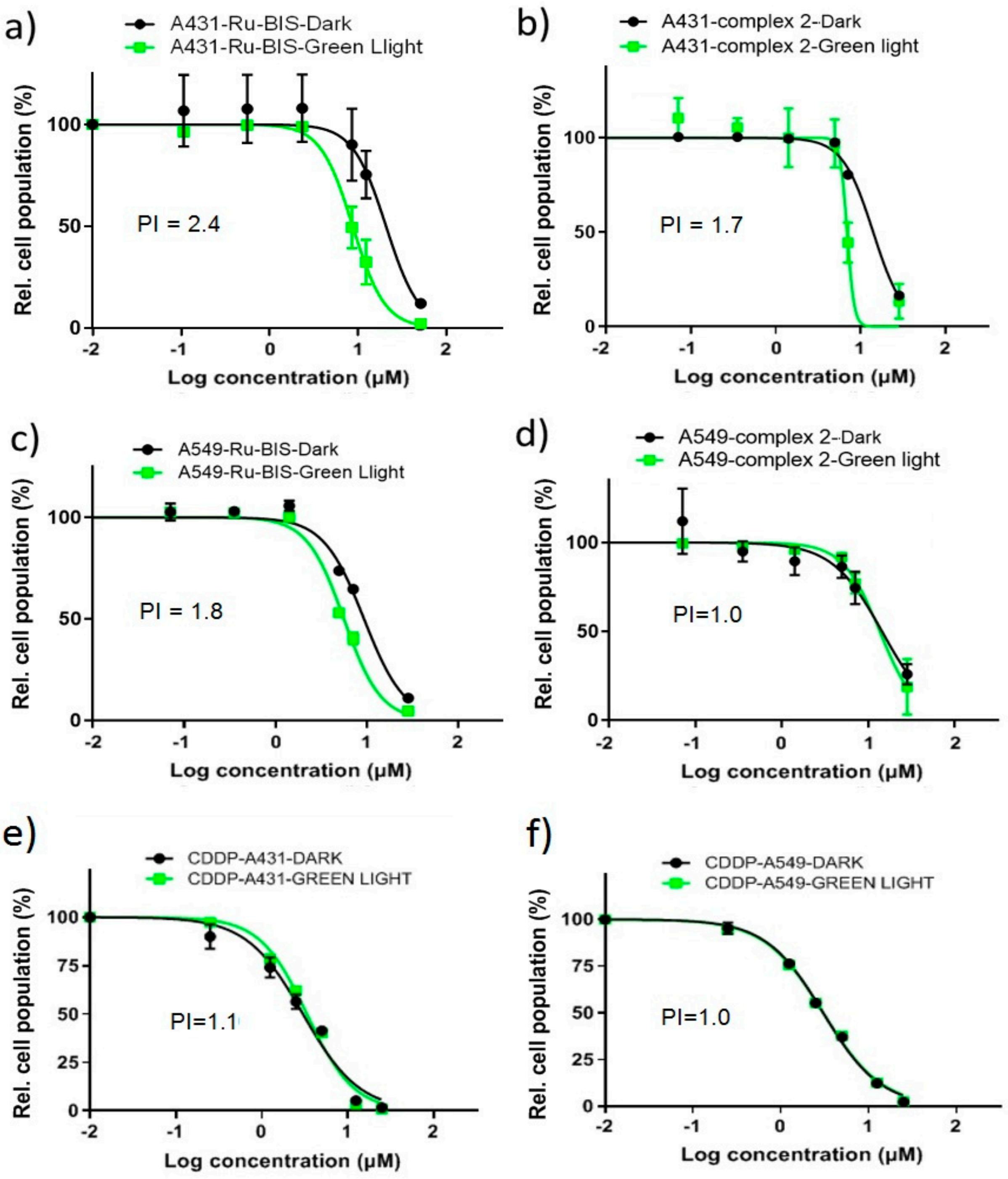

Figure S19. Comparative dose-response curves and calculated phototoxicity index (PI) after 1h irradiation with green light (green data points) and in the dark (black data points) for: A431 cell line in presence of **RuBIS** CPNs (a) or the complex **2** (b); A549 cell line in presence of **RuBIS** CPNs (c) or the complex **2** (d); and cisplatin (CDDP) cytotoxicity dose-response curves for A431 (e) and A549 (f) cell lines. Phototoxicity assay outline: cells were incubated in the dark for 48 h, and the plates were irradiated for 60 min with green light (λ_{exc} = 520 nm, power density 10.92 mW/cm^2, light dose = 39.3 J/cm^2). SRB assay was performed at t=96h. Data points represent the average of triplicates (confidence interval of 95% in M).

3.2 Cytotoxicity of Bis and 1-methylimidazole free ligands

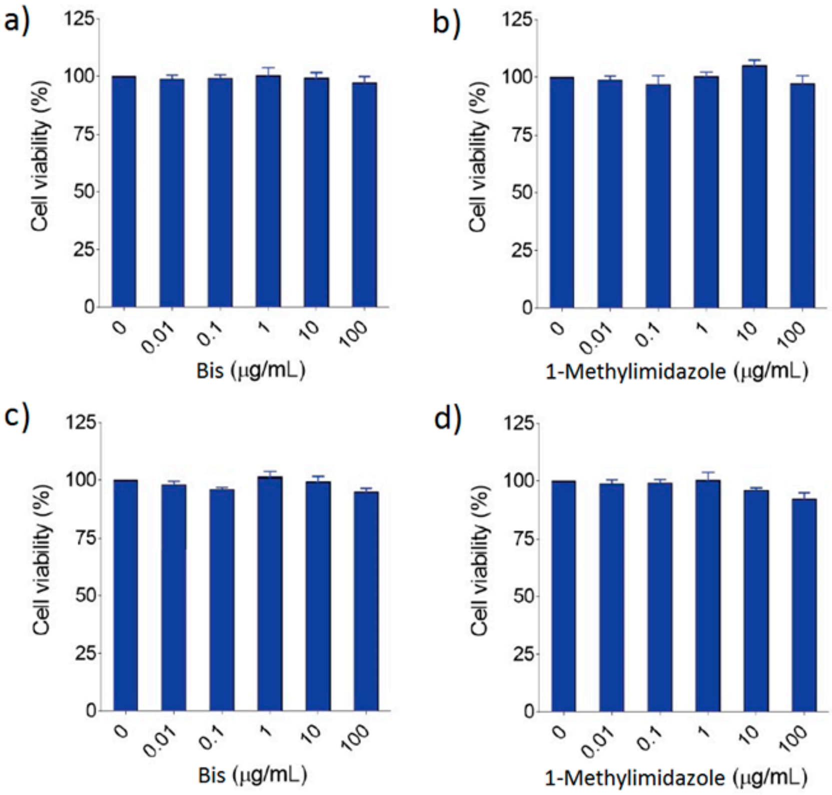

Figure S20. Cell viability studies of Bis and 1-methylimidazole free ligands in a-b) non-malignant human fibroblast cells (1Br3G) and c-d) HeLa cell lines at different concentrations ranging from 0 to 100 μg/mL. Cell viability was evaluated at 48h by a resazurin-based assay using the PrestoBlue cell viability reagent.

3.3 $^1O_2$ studies for RuBIS CPNs and 2 under normoxic conditions with and without green light (520 nm) irradiation

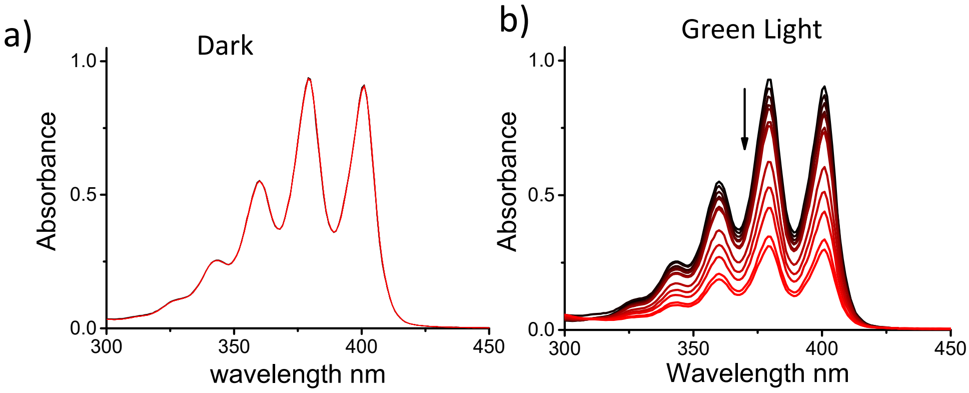

Figure S21. Absorption spectral changes of ABMDMA in the dark (a) or following green light irradiation with the light of dose 39.3 J/cm^2 (b) in the presence of Rose Bengal (0.1 μM).

3.3 Determining the required irradiation time for RuBIS CPNs and complex 2 activation

Mock irradiation experiments were performed in the conditions of the cytotoxicity assay, but in absence of cells, to determine the irradiation time necessary to activate **RuBIS** CPNs and complex **2** in the cytotoxicity assay. The highest concentration (50 μg/mL) of **RuBIS** CPNs was added (200 μL) in transparent 96-well plates. Samples were irradiated for 60 min with green light (λ_{exc} = 520 nm, 39.3 J/cm^2) and absorption changes at 440 and 390 nm for

RuBIS CPNs and complex **2** was followed vs. irradiation time. The complete activation of both compounds was achieved after 60 min of continuous irradiation (light dose 39.3 J/cm^2).

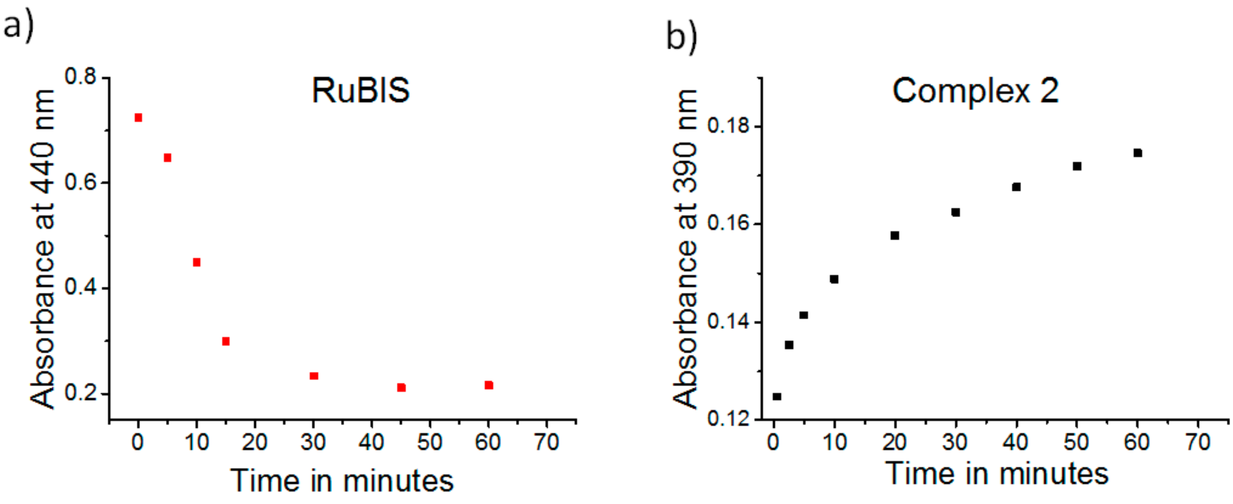

Figure S22. Mock irradiation studies for (a) **RuBIS** CPNs and (b) complex **2** was conducted in cell culturing Opti-MEM media at 37 ºC in air without the cells to know the light intensity and duration of time for the complete activation of these compounds. Conditions: 50 µg/mL of **RuBIS** CPNs or complex **2** (200 µL), light irradiation λ_{exc} = 520 nm, 39.3 J.cm^{-2}.

Reference:

1. van Rixel, V.H.S.; Siewert, B.; Hopkins, S.L.; Askes, S.H.C.; Busemann, A.; Siegler, M.A.; Bonnet, S. Green light-induced apoptosis in cancer cells by a tetrapyridyl ruthenium prodrug offering two trans coordination sites. *Chem Sci* **2016**, *7*, 4922-4929.
